# Phosphoramide Hydrogels as Biodegradable Matrices for Inkjet Printing and Their Nano-Hydroxyapatite Composites

**DOI:** 10.1021/acsami.4c10532

**Published:** 2024-09-19

**Authors:** Mahsa Mostofizadeh, Michael Kainz, Farzaneh Alihosseini, Stephan Haudum, Mostafa Youssefi, Peter Bauer, Iurii Gnatiuk, Oliver Brüggemann, Katja Zembsch, Uwe Rinner, Catarina Coelho, Elena Guillén, Ian Teasdale

**Affiliations:** † Department of Textile Engineering, Isfahan University of Technology, Isfahan 84156-83111, Iran; ‡ Institute of Polymer Chemistry, Johannes Kepler University, Linz 4040, Austria; § Functional Surfaces and Nanostructures, Profactor GmbH, Steyr-Gleink 4407, Austria; ∥ TIGER Coatings GmbH & Co.KG, Wels 4600, Austria; ⊥ Institute of Applied Chemistry, IMC University of Applied Sciences Krems, Piaristengasse 1, Krems 3500, Austria; # FLUIDINOVA, S.A.,Maia 4475-188, Portugal

**Keywords:** nano-hydroxyapatite, phosphoramide, inkjet printing, tissue engineering, additive manufacturing

## Abstract

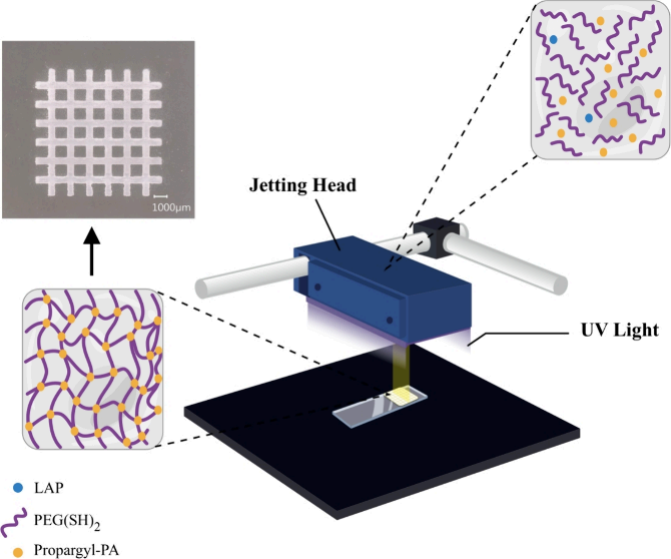

Inkjet printing is a leading technology in the biofabrication of three-dimensional biomaterials, offering digital, noncontact deposition with micron-level precision. Among these materials, hydroxyapatite is widely recognized for its use in bone tissue engineering. However, most hydroxyapatite-laden inks are unsuitable for inkjet printing. To address this, we developed photocurable and biodegradable phosphoramide-based hydrogels containing thiol-functionalized polyethylene glycol via click chemistry. These hydrogels degrade into phosphates, the natural component of bone. The rheological properties of the inks are finely tuned through chemical design to meet the requirements of nanohydroxyapatite composite inks for piezoelectric inkjet printing. We demonstrated their printability using simple geometric patterns, showcasing a versatile and efficient solution for the precise inkjet printing of biomaterial composites.

## 1 Introduction

Three-dimensional (3D) inkjet printing is a powerful additive manufacturing technique for biomaterial fabrication and has promising applications in fields such as drug delivery,^1^ bone tissue engineering,^2^ and the bioprinting of microvessels.^3^ Inkjet printing offers the advantage of high printing speeds and precise digital deposition at the micrometer scale.^4^ Furthermore, unlike most additive manufacturing processes, which are usually restricted to the usage of a single material, it is also ideally suited for fabricating multimaterial constructs.^4^ However, inkjet printing, particularly with multinozzle printheads, is heavily restricted by fluid rheology. For optimal printing, ink viscosity should fall within 3–25 mPa s at temperatures ranging from 20 to 65 °C, with maximum particle agglomerations between 1 and 6 μm required; hence, careful ink design is critical.^5^

Hydroxyapatite (HAp) is a popular bone-graft biomaterial, and due to its compositional similarities to bone, it can enhance cellular activity and induce bone formation.^6,7^ Nanocrystalline hydroxyapatite (nHAp) particles have certain advantages over microsized HAp. Unlike traditional microsized HAp, nHAp demonstrates improved protein adsorption on its larger surface area, influencing cell behavior like adhesion, proliferation, and osteogenic differentiation.^8,9^ Due to their high surface area-to-volume ratio and similarity with the bone mineral, aqueous nHAp pastes are reported to be osteostimulative, promoting the adhesion of bone cells and fast formation of new bone,^10^ and have already been used in intraoperatively bioprinted bone constructs.^11^

In biomedical applications, 3D printing of HAp has gained traction, particularly for creating mechanically rigid structures using HAp ceramic slurries, often employing vat polymerization followed by a sintering process.^12^ Stereolithography has enabled the fabrication of HAp-laden photopolymer hydrogels based on cross-linkable polyethylene glycol (PEG) diacrylate matrices.^13^ Uelhin et al. utilized a single-nozzle pneumatic valve inkjet printer to deposit drops of a nHAp dispersion for anatomical mineralization profiles.^2^ They immobilized the particles on a synthetic polymer using a fibrin hydrogel. Wee et al. employed drop-on-demand printing of HAp-Alginate mixtures to create HAp microspheres with single-nozzle printing and ionic cross-linking.^14^

Until recently, photochemical 3D inkjet printing has been restricted to acrylate-based photopolymers due to the fast photokinetics and low viscosity required for this advanced technology. However, such inks lead to nonbiodegradable photocured polymers that are unsuitable as in vivo tissue regeneration scaffolds and are generally regarded as cytotoxic.^15^ Recently, we developed a platform of amino acid phosphoramidates and studied them as biodegradable photopolymer resins for digital light processing, multiphoton lithography, and 3D inkjet printing.^16−18^ Due to the substitution of amino acids on the phosphate core, these compounds demonstrate varying rates of hydrolysis, degrading to phosphates and amino acids, the main constituents of bone tissue. As these previous materials were all hydrophobic resins, in this study, we have created water-soluble monomers based on phosphoramides, which can undergo cross-linking through UV-initiated thiol-yne chemistry, thereby establishing a versatile platform for tailoring the mechanical, chemical, and degradation properties of hydrogels. We then study water-based nHAp suspensions and their hydrogel composites specifically for precise thin film printing using a multinozzle inkjet printhead.

## 2 Experimental Section

### 2.1 Materials

Unless stated otherwise, all chemicals were purchased from Merck and used without further purification. Prior to use, all solvents were dried with 3 Å molecular sieves and all chemical reactions were performed under an argon (Ar) atmosphere. Hydroxyapatite nanoparticles (nanoXIM·HAp102, FLUIDINOVA, Portugal) dispersed in water composed of 15.0 ± 1.0 wt % rod-like nanoparticles (particle size < 100 nm, with 30 nm average length and 15 nm average width).

### 2.2 Methods

#### 2.2.1 NMR Spectroscopy

All NMR spectra were measured using a Bruker Avance III 300 MHz spectrometer to obtain ^1^H NMR, ^13^C NMR, and ^31^P NMR spectra. Solid-state NMR spectroscopy was conducted on a Bruker Avance III 500 MHz.

#### 2.2.2 Monomer Hydrolysis

Monomer hydrolysis rates were monitored at pH 4.5, 7.4, and 12.0 at 37 °C utilizing 25 mg of each monomer and the corresponding buffer solution (D_2_O:citrate buffer (0.5 M) (1:9) at pH of 4.5; D_2_O:HEPES buffer (1 M) (1:9) for glycine amino acid phosphoramides (Gly-APA) and DMSO:HEPES buffer (1 M) (1:3) for alanine amino acid phosphoramides (Ala-APA) at pH 7.4; D_2_O:NaHCO_3_/Na_2_CO_3_ buffer (0.5M) (1:9) at pH of 12.0). Gly-APA, Ala-APA, propargyl phosphoramide (Propargyl-PA), and allyl phosphorodiamidates (Allyl-PdA) were dissolved in the corresponding buffer solutions, transferred to NMR tubes, and incubated at 37 °C, and their ^31^P NMR spectra were measured periodically. ^31^P NMR monitoring revealed progressive monomer hydrolysis over time, evidenced by the emergence and intensification of new peaks alongside the diminishing of the initial peak.

#### 2.2.3 Rheological Characterization

The rheological measurements were conducted using an Anton Paar MCR 702 rheometer with a double gap geometry and a shear rate from 1 to 1000 s^–1^ at 40 °C. UV rheological measurements were performed on an Anton Paar MCR 502 in PP 10 mm configuration. The bottom plate a UV-permeable quartz disc was used, enabling the direct UV exposure (Solo P lamp 405 nm, 5 W) of the sample.

#### 2.2.4 Preparation of Stabilized nHAp Suspensions

The particles were dispersed mechanically and stabilized electrostatically with the aim of keeping the solid nHAp content as high as possible while tuning the viscosity in unsheared conditions and particle size distribution by reducing agglomerates below 1 μm. Hence, the following preparation steps were applied: (i) ultrasonication (TI-H-10 Elma multifrequency ultrasonic cleaning device, 25 kHz using a cycle of 10 min, and an amplitude of 100%), (ii) centrifugation (Heraeus Thermo Scientific Biofuge Primo, 50 mL centrifugal tube with 40 mL nanoXIM·HAp102, 10 min, 5000 rpm), and (iii) stabilizing nHAp with sodium citrate (HAp/citrate molar ratio of 4) as a dispersant, leading to a stabilized nanoXIM·HAp102. The aim of the use of a dispersant is to enhance repulsive forces among particles and reduce their agglomeration tendency to prepare stable nanosuspensions for inkjet printing.

After centrifugation, the supernatant was removed from the centrifugal tube and the solid content was determined gravimetrically. The sample of the nHAp suspension was weighed into a ceramic crucible and ashed at 900 °C (heating rate 3 °C per min) in a microwave-assisted muffle furnace (Phoenix, CEM) under air. Afterward, the crucible was cooled and weighed to calculate the residue. A lithium metaborate digestion was prepared from this residue, which was dissolved in 7 wt % nitric acid solution. The calcium content of the obtained dissolution was then determined by ICP-OES (iCAP 7000, Thermo Scientific).

The particle size distribution of the nHAp suspension was characterized by a differential sedimentation method using a CPS disc centrifuge, CPS Instruments. The following HAp properties were used for calculation of the particles size distribution: refractive index, 1.64; particle absorption, 0.1; particle nonsphericity, 1.

The filtration characteristics of the samples were evaluated at 22 °C using a filtration rig consisting of a pressurized 0.5 L container at 0.3 bar equipped at the bottom with an outlet where a syringe disc filter (25 mm) could be connected using a luer lock connector. The amount of ink that passes through the 25 mm disc filter (3 μm) before it blocks was recorded.

### 2.3 Preparation of Phosphoramide-Based Hydrogels

All hydrogels were prepared by employing a 60 wt % solution in water of a stoichiometric formulation containing the various phosphoramides, the thiol cross-linker (PEG(SH)_2_), and 1.5 wt % of LAP (lithium-phenyl-2,4,6-trimethylbenzoylphosphinate, based on monomer content) as an initiator. The hydrogels were then created using circular PDMS molds (12 mm diameter and 2 mm thickness) and cured at 365 nm with a Rayonet RPR-200 UV reactor overnight.

The hydrogel inkjet formulation comprised a 30 wt % solution in water of a stoichiometric mixture of ALA-APA and (PEG(SH)_2_), 1.5 wt % of LAP, and 0.5 wt % of pyrogallol (based on monomer content) as an initiator and a stabilizer.

The nHAp/hydrogel formulation comprised a 25 wt % solution in the stabilized nHAp suspension (section 2.2.4) of a stoichiometric mixture of Propargyl-PA and (PEG(SH)_2_), 1.5 wt % of LAP, and 0.5 wt % of pyrogallol was used.

### 2.4 Gel Fraction

The gel fraction of the hydrogels was determined gravimetrically. Standardized dried hydrogel discs (12 mm diameter and 2 mm thickness) were submerged in ethyl acetate (EtOAc) for 16 h to remove the unreacted monomers. After immersion, the discs were dried to a constant weight in a vacuum oven. The gel fraction was then calculated as the weight ratio of the postextraction hydrogels to the initial weight.

### 2.5 Mechanical Properties

DMA measurements were performed on a TA Instruments DMA Q800. For this purpose, dumbbell-shaped specimens (2 mm thick) were fabricated using PDMS molds. The force and displacement data collected during this process enabled the calculation of key material properties like tensile strength and Young’s modulus from the resulting stress–strain curve. The tensile tests were performed at room temperature (RT) using a force ramp of 0.25 N min^–1^ until failure.

### 2.6 Swelling Behavior

To determine the swelling ratio, the cured hydrogels were first dried, and the weight was recorded and considered as the initial dry weight. Subsequently, these discs were immersed in buffer solutions with pH values of 4.5 and 7.4 at 37 °C. After a specified time interval, each disc was removed from the buffer solution, and excess buffer was taken using filter paper. The weight of each disc was then recorded and considered as the secondary weight. The swelling ratio was then calculated as the difference between the secondary weight and initial dry weight divided by the initial dry weight of hydrogel.^19^ All measurements were done in triplicates.

### 2.7 Hydrogel Degradation

The degradation behavior of the hydrogels was evaluated by monitoring their mass loss over time. Standardized disc-shaped samples were first extracted with EtOAc to extract any unreacted monomers, followed by drying the discs in a vacuum oven until a constant weight. This weight was recorded as the initial dry weight. Subsequently, the samples were submerged in buffer solutions at pH 4.5 and 7.4 at 37 °C. At predetermined time intervals, the samples were retrieved from the buffer solutions, rinsed thoroughly with water, and dried again in a vacuum oven until a constant weight was reached (secondary dry weight). The degradation of the hydrogels was then assessed by the mass percentage of the secondary dry weight to that of the initial dry weight.^20^ All measurements were done in triplicates.

### 2.8 Inkjet Printing

Inkjet printing was done on a Dimatix DMP2800 with a 10 pL cartridge and 16 nozzles. Printing temperature was set to 45 °C. The printing resolution of the patterns was set between 635 and 1270 dpi. Printing was conducted on microscopic glass slides (dimensions: 25 × 75 mm, thickness: 1 mm), with the substrate temperature maintained at 25 °C.

### 2.9 Statistical Analysis

All measurements were performed in triplicate, and the results were presented as mean ± standard deviation (SD). Statistical analysis was conducted using one-way analysis of variance (ANOVA) with SPSS 16 software, and a *p*-value of less than 0.05 was considered as the significant difference.

### 2.10 Chemical Synthesis

#### 2.10.1 Poly(ethylene glycol)-dithiol (PEG(SH)_2_)

The synthesis of poly(ethylene glycol)-dithiol (PEG(SH)_2_) was adopted from a reported procedure. Briefly, polyethylene glycol (*M*_w_ = 1000) (20 g, 20 mmol, 1.0 equiv) and *p*-toluenesulfonic acid monohydrate (133 mg, 0.7 mmol, 0.04 equiv) were dissolved in 200 mL of toluene and mercaptopropionic acid (4.35 g, 41 mmol, 2.05 equiv) was added. The reaction mixture was then refluxed at 140 °C under Dean–Stark conditions for 16 h (Scheme S1a). Then, the solvent was removed under a reduced pressure. The remaining residue was dissolved in EtOAc and washed with brine thrice. Eventually, the organic phase was dried with magnesium sulfate (MgSO_4_) and the solvent was removed under reduced pressure, yielding the product.

PEG(SH)_2_ (white viscous wax, yield 82%): ^1^H NMR (300 MHz, CDCl_3_, δ/ppm): 4.28 (t (*J* = 4.8 Hz), 4H, CH_2_CH_2_OCO), 3.91–3.39 (m, 90H, OCH_2_CH_2_O), 2.83–2.75 (m, 4H, CH_2_), 2.72–2.67 (m, 4H, CH_2_), 1.69 (t (*J* = 8.2 Hz), 2H, SH).

#### 2.10.2 Synthesis of Boc-Protected Propargyl Amino Acid Substituents

The Boc-protected propargyl amino acid substituents were synthesized, following the literature procedures.^16^ Briefly, the Boc-protected amino acid (10.0 g, 1.0 equiv) was dissolved in 260 mL of DMF. Potassium carbonate (K_2_CO_3_, 1.2 equiv) was added, and the reaction mixture was cooled using an ice bath. Then, propargyl bromide (1.0 equiv) was added dropwise. After complete addition, the mixture was stirred at RT for 17 h (Scheme S1b). Then, the reaction mixture was filtered, and the solvent was removed under reduced pressure. The residue was redissolved in EtOAc, washed with brine thrice, and dried with MgSO_4_, and the solvent was evaporated under reduced pressure.

Glycine (R = H) (viscous liquid, yield 90%): ^1^H NMR (300 MHz, CDCl_3_, δ/ppm): 4.92 (s, 1H, NH), 4.68 (d (*J* = 2.5 Hz), 2H, −CH_2_), 3.90 (d (*J* = 5.6 Hz), 2H, CH_2_), 2.43 (t (*J* = 2.5 Hz), 1H, CH), 1.39 (s, 9H, CH_3_).

Alanine (R = CH_3_) (viscous liquid, yield 95%): ^1^H NMR (300 MHz, CDCl_3_, δ/ppm): 5.13 (s, 1H, NH), 4.70–4.58 (m, 2H, −CH_2_), 4.24 (s, 1H, CH–CH_3_), 2.44 (t (*J* = 2.5 Hz), 1H, CH), 1.35 (s, 9H, CH_3_), 1.31 (d (*J* = 7.2 Hz), 3H, CH–CH_3_).

#### 2.10.3 Synthesis of Propargyl Amino Acid HCl Substituents

The corresponding Boc-protected propargyl amino acid substituent (20 g, 1.0 equiv) was dissolved in 50 mL of dry dioxane. Subsequently, 4 M HCl in dioxane (2.15 equiv) was added dropwise, and the reaction mixture was reacted at RT for 16 h (Scheme S1c). The resulting precipitate was filtered off and washed thoroughly with dry diethyl ether. Finally, the precipitate was dried under a vacuum.

Glycine (R = H) (white powder, yield 90%): ^1^H NMR (300 MHz, CDCl_3_, δ/ppm): 8.59 (s, 3H, NH_3_^+^), 4.85 (d (*J* = 2.5 Hz), 2H, CH_2_), 3.84 (s, 2H, CH_2_), 3.69 (t (*J* = 2.5 Hz), 1H, CH).

Alanine (R = CH_3_) (white powder, yield 88%): ^1^H NMR (300 MHz, DMSO_d6_, δ/ppm): 8.75 (s, 3H, NH_3_^+^), 4.90–4.80 (m, 2H, CH_2_), 4.11 (q (*J* = 7.2 Hz), 1H, CH_3_–CH), 3.70 (t (*J* = 1.4 Hz), 1H, CH), 1.44 (d (*J* = 7.2 Hz), 3H, CH_3_).

#### 2.10.4 Synthesis of Glycine and Alanine Amino Acid Phosphoramides (Gly-APA and Ala-APA)

Glycine amino acid phosphoramide (Gly-APA) and alanine amino acid phosphoramide (Ala-APA) were synthesized following the literature procedure.^22^ The corresponding propargyl amino acid HCl salt (10.0 g, 3.03 equiv) was dispersed in 200 mL of acetonitrile (ACN). Triethylamine (Et_3_N, 6.3 equiv) was then added, the reaction mixture was cooled to 0 °C, and phosphoryl bromide (1.0 equiv) dissolved in ACN was added dropwise. After complete addition, the mixture was reacted at 55 °C for 15 h (Scheme S 1d). The resulting precipitate was filtered off, and the solvent was removed under reduced pressure. The residue was redissolved in EtOAc and washed once with ammonium chloride solution (NH_4_Cl) and brine. Finally, the organic phase was dried with MgSO_4_ and the solvent was evaporated under reduced pressure.

Gly-APA (R = H) (brown viscous liquid, yield 95%): ^1^H NMR (300 MHz, CDCl_3_, δ/ppm): 4.73 (d (*J* = 2.4 Hz), 6H, CH_2_), 3.86–3.78 (m, 6H, CH_2_), 3.58–3.47 (m, 3H, NH), 2.50 (t (*J* = 2.5 Hz), 3H, CH).

^31^P NMR (121 MHz, CDCl_3_, δ/ppm): 15.2.

^13^C NMR (75 MHz, CDCl_3_, δ/ppm): 171.8, 77.2, 75.6, 52.7, 42.6.

HR-ESI-MS (positive mode): *m*/*z* 384.1001 [M + H]^+^, 767.1926 [2 M + H]^+^.

Ala-APA (brown viscous liquid, yield 90%): ^1^H NMR (300 MHz, CDCl_3_, δ/ppm): 4.72–4.58 (m, 6H, CH_2_), 4.20–3.88 (m, 3H, NH), 3.22 (t (*J* = 9.6 Hz), 3H, CH), 2.45 (t (*J* = 2.4 Hz), 3H, CH), 1.43 (d (*J* = 7.2 Hz), 9H, CH_3_).

^31^P NMR (121 MHz, CDCl_3_, δ/ppm): 11.1.

^13^C NMR (75 MHz, CDCl_3_, δ/ppm): 173.9, 77.2, 75.4, 52.6, 49.6, 20.7.

HR-ESI-MS (positive mode): *m*/*z* 426.1470 [M + H]^+^, 851.2848 [2 M + H]^+^.

#### 2.10.5 Synthesis of Propargyl Phosphoramide (Propargyl-PA)

Phosphoryl bromide (10.0 g, 34.9 mmol, 1.0 equiv) was dissolved in 160 mL of dry acetonitrile ACN, and Et_3_N (16.6 mL, 119 mmol, 3.4 equiv) was added. The mixture was cooled to 0 °C, and propargylamine (6.9 mL, 108 mmol, 3.1 equiv) was added dropwise. After complete addition, the mixture was reacted at 35 °C for 16 h (Scheme S1e). The resulting precipitate was filtered off, and the solvent was removed under reduced pressure. The residue was redissolved in EtOAc and washed twice with brine. Finally, the organic phase was dried with MgSO_4_ and the solvent was evaporated under reduced pressure to yield the product as a brown viscous liquid (yield 89%).

^1^H NMR (300 MHz, CDCl_3_, δ/ppm): 3.87–3.75 (m, 6H, CH_2_), 3.19–2.80 (m, 3H, NH), 2.29 (t (*J* = 2.5 Hz), 3H, CH).

^31^P NMR (121 MHz, CDCl_3_, δ/ppm): 15.4.

^13^C NMR (75 MHz, CDCl_3_, δ/ppm): 82.2, 71.3, 30.5.

HR-ESI-MS (positive mode): *m*/*z* 210.0821 [M + H]^+^, 419.1569 [2 M + H]^+^.

#### 2.10.6 Synthesis of Allyl Phosphorodiamidate (Allyl-PdA)

Ethyl dichlorophosphate (10.0 g, 61.4 mmol, 1.0 equiv) was dissolved in 150 mL of dry tetrahydrofuran (THF), and Et_3_N (19.6 mL, 142 mmol, 2.3 equiv) was added. The mixture was cooled to 0 °C, and allylamine (9.7 mL, 129 mmol, 2.1 equiv) was added dropwise. After complete addition, the mixture was reacted at RT for 15 h (Scheme S1f). The resulting precipitate was filtered off, and the solvent was removed under reduced pressure. The residue was redissolved in EtOAc and washed twice with brine. Finally, the organic phase was dried with MgSO_4_ and the solvent was evaporated under reduced pressure to yield the product as a yellow solid (yield 91%).

^1^H NMR (300 MHz, CDCl_3_, δ/ppm): 5.95–5.59 (m, 2H, CH), 5.21–4.91 (m, 4H, CH_2_), 3.94 (quint. (*J* = 3.9 Hz), 2H, CH_2_), 3.58–3.29 (m, 4H, CH_2_), 2.99–2.67 (m, 2H, NH), 1.21 (t (*J* = 7.1 Hz), 3H, CH_3_).

^31^P NMR (121 MHz, CDCl_3_, δ/ppm): 16.0.

^13^C NMR (75 MHz, CDCl_3_, δ/ppm): 136.7, 114.9, 60.8, 43.5, 16.2.

HR-ESI-MS (positive mode): *m*/*z* 205.1145 [M + H]^+^, 409.2214 [2 M + H]^+^.

## 3 Results and Discussion

### 3.1 Chemical Design and Synthesis of Monomer Library

**Figure 1:**
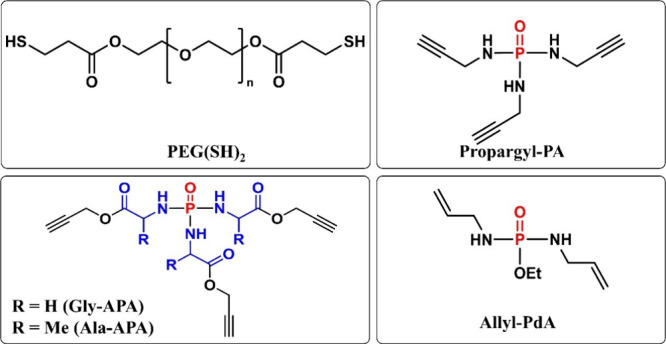
Chemical structures of the used monomers.

Recently, we reported alkynyl amino acid phosphorodiamidates (APdAs)^16^ and amino acid phosphoramides (APAs)^22^ with controlled degradation for thiol-yne photopolymerization. Utilizing hydrophobic ester- or silyl ether-based thiols as comonomers led to hydrophobic materials. Herein, we expand the scope of amino acid phosphoramides to hydrogels. For this, we synthesized the novel amino acid phosphoramides (APAs), Gly-APA,Ala-APA and nonamino acid phosphoramides, Propargyl-PA and Allyl-PdA monomers (Figure 1). The phosphoramides Gly-APA, Ala-APA, and Propargyl-PA were accessible through the straightforward nucleophilic substitution reaction of phosphoryl bromide with the corresponding amine or amino acid substituent. Similarly, Allyl-PdA was synthesized by the reaction of ethyl dichlorophosphate with allylamine. These phosphoramide monomers were then copolymerized with a bismercapto poly(ethylene glycol) (PEG(SH)_2_) in aqueous solution by thiol-yne photopolymerization to create hydrogels. The hydrophilic PEG dithiol cross-linker was synthesized from PEG 1000 by Fischer esterification with 3-mercaptopropionic acid in the presence of *p*-toluenesulfonic acid, as previously reported in the literature. The chemical reaction schemes of all monomers are depicted in the Supporting Information. All monomers were characterized by ^1^H NMR, ^13^C NMR, ^31^P NMR spectroscopy, and mass spectrometry (Figures S1–S18).

#### 3.1.1 Monomer Hydrolysis

**Figure 2:**
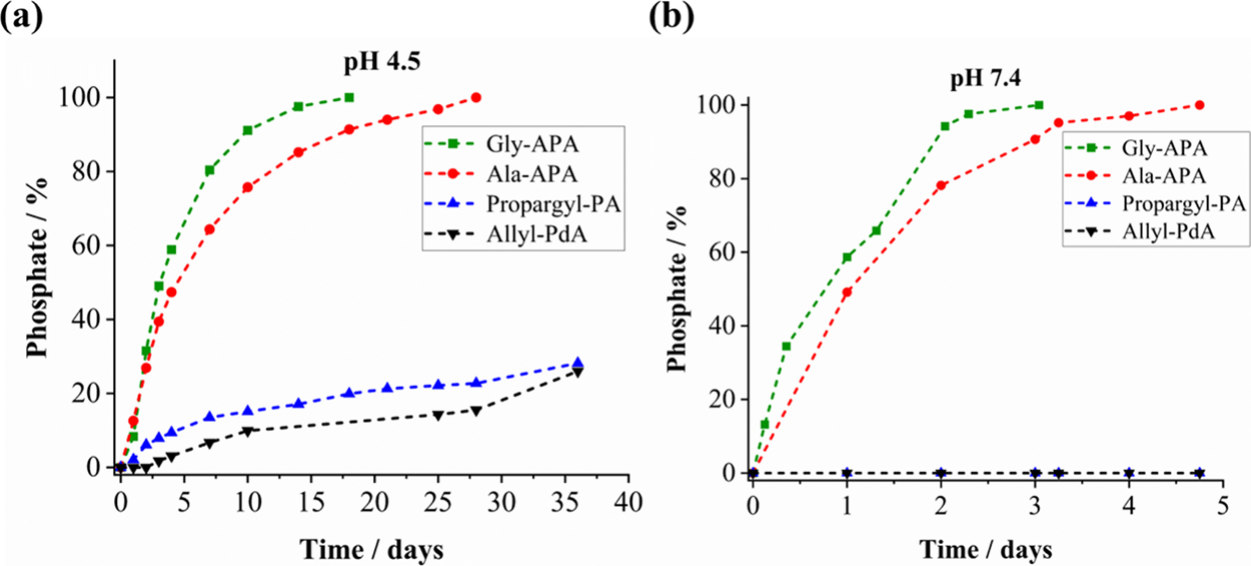
Hydrolysis of the synthesized monomers was monitored by ^31^P NMR spectroscopy. Comparison of the hydrolysis rates of Gly-APA, Ala-APA, Propargyl-PA, and Allyl-PdA monomers at 37 °C in (a) pH 4.5 and (b) pH 7.4.

To aid the subsequent hydrogel design, the hydrolytic stability of the monomers was investigated at 37 °C and a pH of 4.5, 7.4, and 12.0. During hydrolysis of the monomers, a shift in the chemical resonance of the phosphorus atom is observable by ^31^P NMR spectroscopy, thereby enabling us to follow the hydrolysis process (Figure S19b). For this purpose, the monomers were dissolved in aqueous buffer systems (section 2.2.2) and stored at 37 °C, and ^31^P NMR spectra were recorded at regular intervals. While at pH 7.4, the nonamino acid containing Propargyl-PA and Allyl-PdA showed no sign of hydrolysis, the APAs (Gly-APA and Ala-APA) exhibited a rapid hydrolysis (Figure 2b), much faster than the previously reported structurally similar APdAs.^16^ This is attributed to the absence of the ethoxy substituent, which provides a stabilizing effect in APdAs. A similar trend was observed at pH 12.0, with APAs undergoing even faster hydrolysis while the nonamino acid-containing phosphoramides remained stable for at least 30 days (Figure S19a). This observation underlines the significantly higher hydrolytic lability of the P–N bond of amino acid phosphoramides compared to their nonamino acid counterparts.^23^ As expected and aligned with the literature,^23^ the hydrolysis of Propargyl-PA and Ally-APdA was accelerated at more acidic conditions of pH 4.5, and the monomers exhibited a slow but steady hydrolysis (Figure 2a). Interestingly, the hydrolysis of the APAs was slower at pH 4.5 than at pH 7.4 and 12.0, indicating a direct nucleophilic attack of water (hydroxide) on the phosphorus atom as the predominant hydrolysis mechanism. Within the APAs, Gly-APA exhibited a faster hydrolysis than Ala-APA under all pH conditions, a behavior also observed for amino acid phosphoramidates.^16,17^ This can be attributed to the additional methyl group of the Ala substituent, which shields the phosphorus atom from the nucleophilic attack of water, thereby hampering hydrolysis.

### 3.2 Photopolymerization

**Figure 3:**
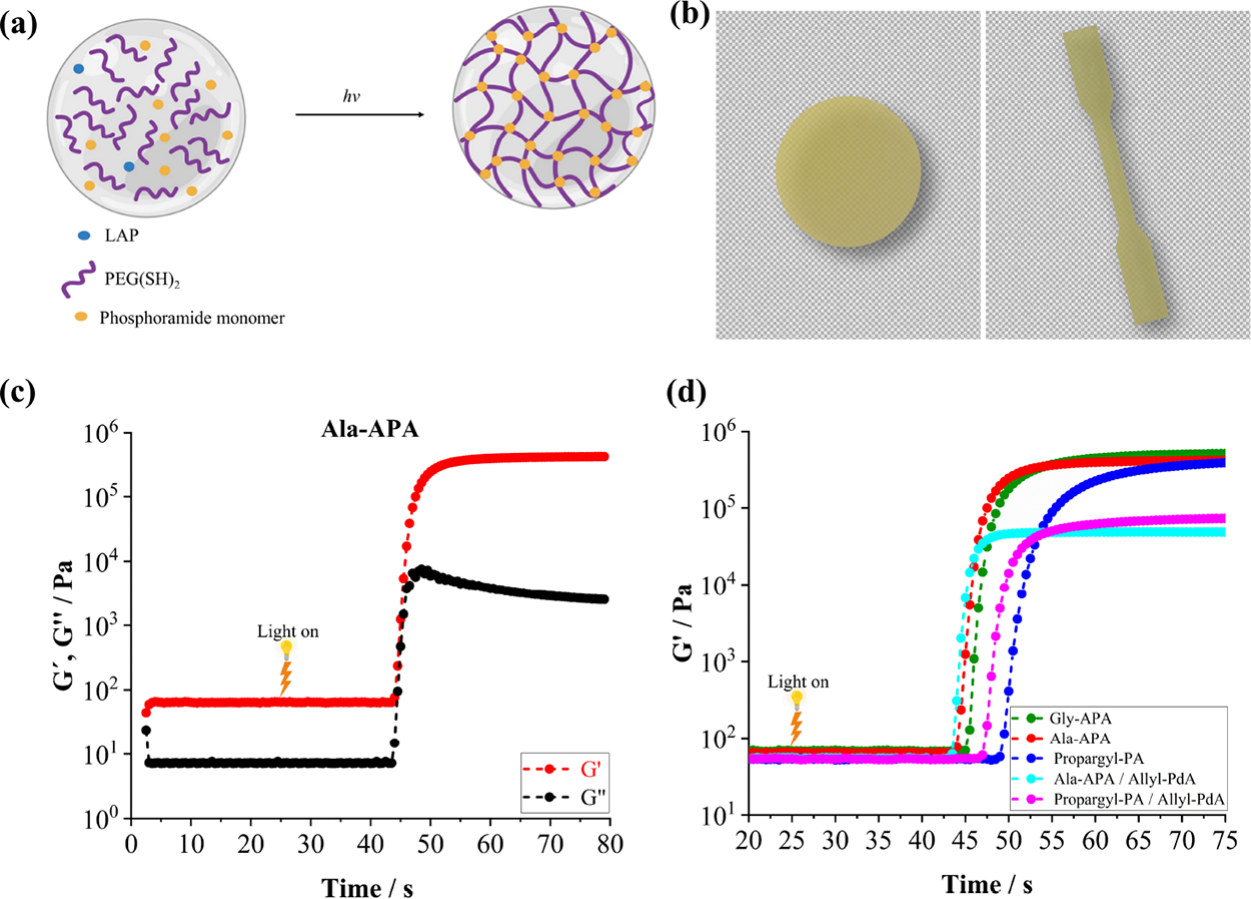
(a) Schematic representation of the thiol-yne reaction used for photopolymerization of phosphoramide-based monomers. (b) Preparation of Ala-APA hydrogels with PDMS molds: This image shows the Ala-APA hydrogels formed after photopolymerization. Circular hydrogels were used for swelling and degradation studies, while dumbbell-shaped hydrogels were used for tensile strength evaluation (the diameter of circular hydrogel is 1.1 cm and the length of dumbbell-shaped hydrogel is 3.4 cm). (c) Rheological monitoring during UV irradiation of the Ala-APA curing solution. (d) The comparison of the storage moduli (*G*′) during UV irradiation of Gly-APA, Ala-APA, Propargyl-PA, Ala-APA/Allyl-PdA, and Propargyl-PA/Allyl-PdA curing solutions over time. UV exposure was initiated 25 s after the onset of the measurements.

The phosphoramide monomers were photopolymerized by thiol-yne chemistry with stoichiometric amounts of PEG(SH)_2_ to form hydrogels (Figure 3a,b). This reaction has been previously studied for the preparation of photopolymers using organic alkynes,^24,25^ acrylates,^26,27^ and vinyl esters^28^ for a wide range of biomaterial applications. The conversion of the hydrogel formulation was investigated by using ^13^C NMR spectroscopy (shown for Ala-APA in Figure S20). This enabled us to follow the thiol-yne reaction, and the disappearance of the Ala-APA triple bond signals (75.6 and 77.2 ppm) indicated a successful polymerization. Additionally, the curing kinetics were examined by UV rheology measurements (Figure 3c,d and Figure S21). During the photopolymerization of the hydrogels and the onset of polymer chain cross-linking, the storage modulus (*G*′) continuously grows and eventually surpasses the loss modulus (*G*″), thereby indicating the formation of a cross-linked hydrogel. The average gelation time (cross over point of *G*′ and *G*″) of the hydrogel formulations was 26 s as shown in Figure 3d and Figure S21. Additionally, the gel fraction of the cured hydrogels was determined to be above 90%, indicating a high conversion and low amount of residual monomers (Figure S22).

### 3.3 Hydrogel Characterization

The mechanical properties of the resulting hydrogels were examined and are presented in Table 1. The careful adjustment of the formulations enabled us to tune the tensile strength, modulus, and yield strain across a broad range. Accordingly, by introducing the bifunctional allyl functionalized Allyl-PdA as a chain extender, we could reduce the cross-linking density and hence create a softer and more flexible material with lower modulus and higher yield strain (Table 1: Propargyl-PA/Allyl-PdA and Ala-APA/Allyl-PdA). Within the trifunctional phosphoramides, the APAs had a slightly higher modulus, reasoned by stronger molecular interactions of the amino acid substituents (Gly-APA, Ala-APA) compared to the amine substituent (Propargyl-PA). Interestingly, the yield strain of the Ala-APA containing hydrogels was significantly greater than those of the other hydrogels.

**Table 1:** Mechanical Properties of Phosphoramide-Based Hydrogels

**hydrogel**	**state**	***E* (MPa)**	**σ (MPa)**	**ε (%)**
Gly-APA	unswollen	0.43 ± 0.09	3.07 ± 0.05	21.69 ± 3.26
Gly-APA	swollen	0.09	1.73 ± 0.13	6.71 ± 0.67
Propargyl-PA	unswollen	0.33 ± 0.13	2.17 ± 0.14	25.26 ± 10.73
Propargyl-PA	swollen	0.03 ± 0.01	1.36 ± 0.23	7.76 ± 5.83
Ala-APA	unswollen	0.58 ± 0.07	2.41 ± 0.09	42.86 ± 6.69
Ala-APA	swollen	0.11 ± 0.08	1.25 ± 0.17	14.92 ± 7.88
Propargyl-PA/Allyl-PdA	unswollen	0.12 ± 0.04	0.6 ± 0.1	49.09 ± 14.53
Ala-APA/Allyl-PdA	unswollen	0.25 ± 0.07	0.66 ± 0.03	128.63 ± 95.49

**Figure 4:**
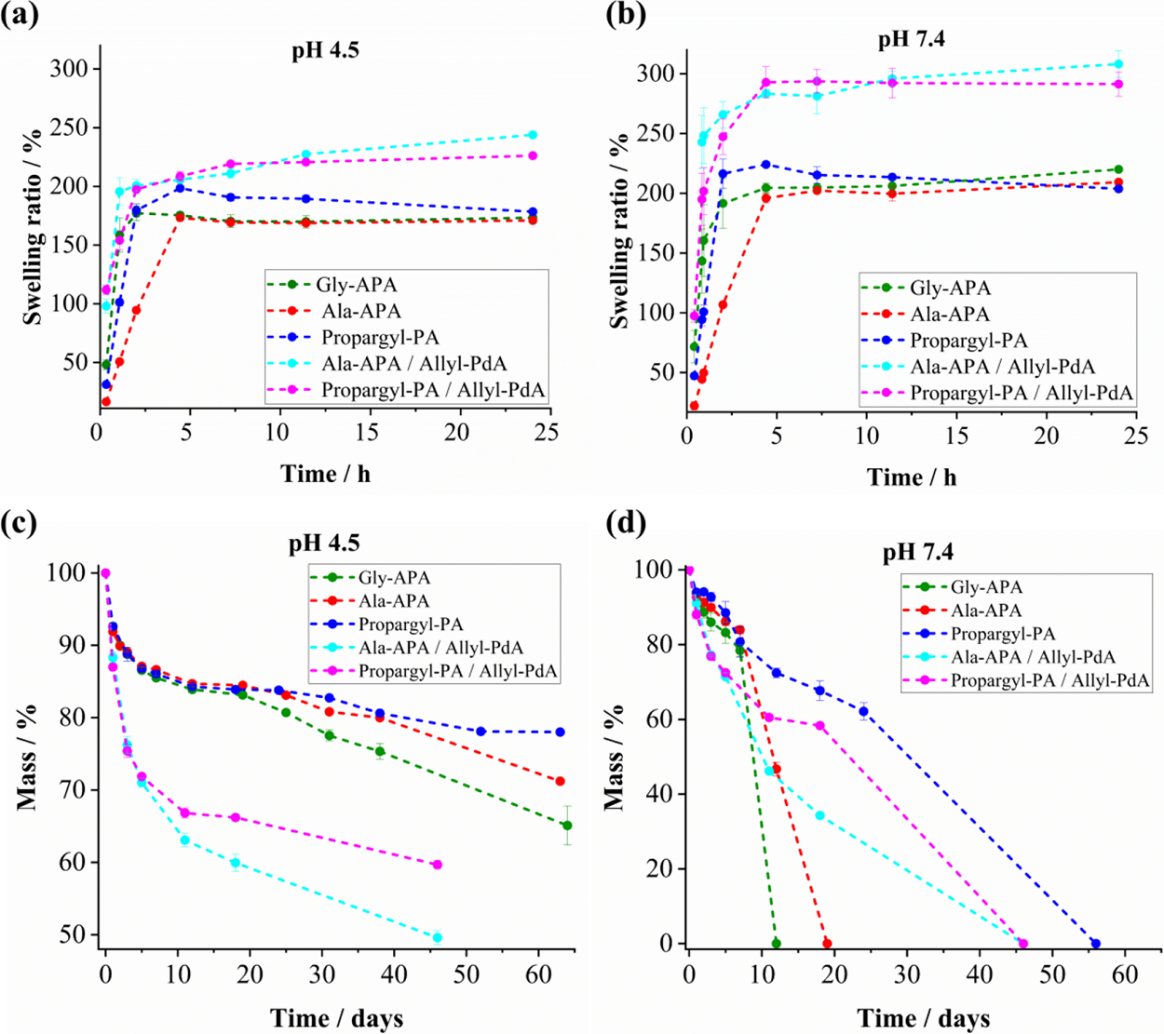
Swelling ratio of phosphoramide-based hydrogels at 37 °C: (a) pH 4.5 and (b) pH = 7.4. Biodegradation of phosphoramide-based hydrogels at 37 °C: (c) pH = 4.5 and (d) pH = 7.4.

The swelling of the hydrogels was evaluated at pH of 4.5 and 7.4 over 24 h (section 2.6). Healthy tissues maintain a neutral pH of around 7.4, but bone resorption involves a localized acidic environment (≤pH 4.5) due to the solubility of hydroxyapatite at this lower pH. Therefore, the hydrogel characterizations were performed at pH of 4.5 and 7.4.^29^ Upon immersion in water, all hydrogels increasingly swelled until they reached an equilibrium after 24 h (Figure 4a,b). The swelling-increasing trend observed at a pH of 7.4 aligns with the water contact-angle measurement results, indicating that samples with a higher swelling ratio exhibit a lower contact angle (Figure S23). The Allyl-PdA containing hydrogels exhibited the highest swelling at both pH values (>200%, Figure 4a,b), which is attributed to their lower cross-linking density (compared to APAs) facilitating the penetration and absorption of water molecules. All other hydrogels (Gly-APA, Ala-APA, and Propargyl-PA) showed a similar swelling in the range of 150% at pH 4.5 and 200% at pH 7.4 (*p* > 0.05).

### 3.4 Bulk Degradation

The bulk degradation behavior of the hydrogels was evaluated by mass loss studies at pH 4.5 and 7.4 at 37 °C (section 2.7). In accordance with monomer hydrolysis data (Figure 2), the APA hydrogels (Gly-APA and Ala-APA) exhibited a faster degradation at pH 7.4 than at pH 4.5. While only losing 29–35% of their original mass at pH 4.5 in 64 days, at pH 7.4, the hydrogels completely degraded within 12 (Gly-APA) and 19 (Ala-APA) days (Figure 4c,d). Despite showing a similar pH-dependent trend, Ala-APA/Allyl-PdA hybrid hydrogels (Figure 4c) degraded considerably faster than the pure APA hydrogels at pH 4.5. This faster hydrolysis is most likely due to the higher swellability of these hydrogels. Interestingly and in contrast to the monomer hydrolysis, the degradation of the nonamino acid containing Propargyl-PA hydrogels (Propargyl-PA and Propargyl-PA/Allyl-PdA) was faster at pH 7.4 than at pH 4.5 (Figure 4c,d). This observation is attributed to a faster hydrolysis of the PEG(SH)_2_ thiol ester component at pH 7.4 compared to pH 4.5, thereby contributing to an overall faster mass loss of the hydrogels.

### 3.5 Inkjet Printing

#### 3.5.1 Hydrogel

The printability of the pristine hydrogel was demonstrated by printing two single-layer structures, namely, a face-centered structure (dimensions: 10.72 mm × 10.72 mm; line width: 720 μm) and a mesh structure (dimensions: 10 mm × 10 mm; line width: 520 μm), in order to study the conditions for the fabrication of patterns and continuous layers with different geometries.

**Figure 5:**
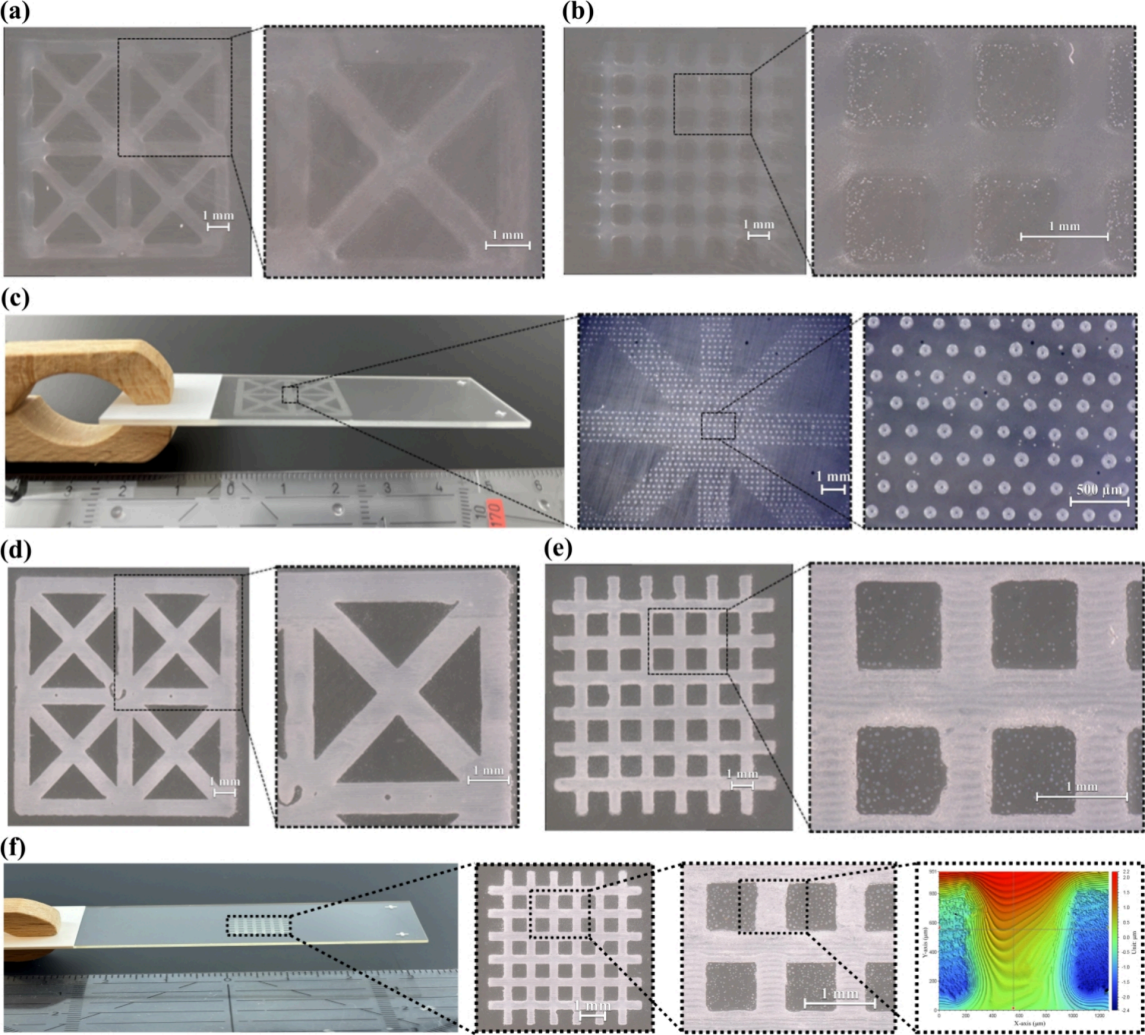
(a, b) Optical microscope image of the inkjet printed Ala-APA hydrogel with (a) a face-centered structure and (b) mesh structure. The right image provides a magnified view of the specified area in the left image (scale bar = 1 mm). (c) Inkjet printed stabilized nHAp suspension. The middle image presents a magnified view of the specified area in the left image, while the right image offers a magnified view of the specified area in the middle image. Inset middle: Scale bar = 1 mm. Inset right: Scale bar = 500 μm. (d–f) Optical microscope image of the inkjet printed nHAp-PA composite. (d) Single-layer face-centered structure. (e) Single-layer mesh structure (f) Four-layer mesh structure. The two middle images provide magnified views of the specified areas in the adjacent left images (scale bar = 1 mm). The right image shows the interpore line surface in the specified area of the adjacent left image, as observed in the 3D-printed mesh structure using a 3D optical profiler.

Ala-APA formulation was chosen due to its favorable swelling and degradation profile. This formulation exhibited a viscosity of 3.8 mPa s at 40 °C, which falls within the optimal range for inkjet printing processes. The results demonstrated that the Ala-APA hydrogel formulation with a concentration of 30 wt % exhibits favorable inkjet printing properties concerning viscosity, jetting, and droplet formation, producing uniform layers and sufficient shape fidelity. Figure 5a,b illustrates printed models using the Ala-APA hydrogel. Each layer was cured with four passes at 1.5 m min^–1^ on a DYMAX UVC conveyor with an Fe-doped mercury lamp. The printing resolution set for this experiment was 1270 dpi.

#### 3.5.2 Inkjet Printing with nHAp

Subsequently, a water-based nHAp suspension ink was developed from a commercially available nHAp (nanoXIM·HAp102).^30−32^ To achieve this, we dispersed the nanoparticles mechanically and stabilized them electrostatically for the preparation of a particle-laden ink for inkjet printing with the aim of keeping the solid nHAp content as high as possible while reducing the viscosity in unsheared conditions and reducing agglomerates below 1 μm. The TEM images of nHAp and its dispersion within the composites are provided in Figure S24. Viscosity measurements showed a highly shear-thinning raw material (Figure S24). The multistep preparation procedure reduces shear thinning, and with the addition of sodium citrate, the viscosity of the nHAp suspension even approached Newtonian behavior. A notable reduction in detected peaks in the particle size distribution was observed after every preparation step (Table S1). In addition, the filterability was significantly improved. The final solid content of nHAp was determined to 10.72 wt %. Prior to printing, jetting behavior and drop formation was observed. Drop formation and the used waveform are depicted in Figure S25. Sodium citrate was selected as a dispersant because of the high levels of citrate in bone,^33^ where citrate might act as a native dispersant by mobilizing calcium transport.^34,35^ Its potential to act as an effective dispersant in stabilizing calcium phosphate (CaP) suspensions by increasing the negative surface charge of the CaP particles and consequently increasing the repulsive interparticle forces, hence preventing them from aggregating was already investigated.^36,37^

The inkjet printability of the stabilized nHAp suspension was demonstrated by printing a single-layer structure (Figure 5c). Additionally, we intentionally reduced the resolution to print nonuniform layers, allowing us to observe the formation of a dot pattern. Such models might be particularly relevant for printing gradients and transition zones. A face-centered structure was chosen, and the resolution was set to 635 dpi.

#### 3.5.3 Inkjet Printing with nHAp Hydrogel Composite

We then created an nHAp/Propargyl-PA hydrogel composite formulation. The Propargyl-PA monomer was selected for these composites due to its longer hydrolytic stability in the presence of the sodium citrate base (see Figure S19). Our investigations revealed that the stabilized nHAp suspension containing Propargyl-PA hydrogel at a concentration of 25 wt % demonstrated optimal viscosity (3.5 mPa s) and stability at 40 °C for inkjet printing. The nHAp solid content in the nHAp hydrogel composite was therefore 8.04 wt %. As showcased in Figure 5d–f, the single-layer face-centered and mesh structures as well as the four-layers mesh structure were successfully printed. Furthermore, incorporating nHAp significantly improved the mechanical properties of the Propargyl-PA hydrogel. While the low 25 wt % polymer concentration only gave weak gels, the modulus, tensile strength, and yield strain were measured to be 10.62 ± 5.42, 0.43 ± 0.08, and 35% ± 0.14, respectively, for the nHAp hydrogel composite. These observations collectively suggest the potential of the nHAp/Propargyl-PA hydrogel composite for inkjet printing applications (the chosen printing resolution of the pattern was 1270 dpi).

## 4 Conclusions

This study describes the chemical design, synthesis, and characterization of a series of water-soluble phosphoramide-based photomonomers. Notably, the incorporation of amino acid substituents in these compounds imparts varying rates of hydrolysis, leading to the formation of phosphates and amino acids, essential constituents of bone tissue. Upon photopolymerization with PEG(SH)_2_, these monomers facilitate the formation of hydrogels, the swelling behavior, the mechanical attributes, and the rheological characteristics of which were systematically investigated. By judiciously selecting monomeric constituents, the physical properties of the resultant hydrogels could be finely modulated, leveraging a synergistic interplay between chemical structure and local environmental factors. Furthermore, the versatility of these hydrogels was demonstrated through their amenability to inkjet printing, employing straightforward geometric patterns. Moreover, an innovative nanohydroxyapatite (nHAp) containing ink formulation was devised specifically for piezoelectric inkjet printing applications. This approach underscores an adaptable strategy for the precise and scalable fabrication of nHAp composites within biodegradable matrices, and future work will look to develop them toward biomaterial applications.

## Supporting Information Available

Additional data for polymer characterization (DOCX)
