# Supplementary material for: Phosphoramide Hydrogels as Biodegradable Matrices for Inkjet Printing and Their Nano-Hydroxyapatite Composites
Source: ACS Appl Mater Interfaces. 2024 Sep 19;16(39):52902–10. doi: 10.1021/acsami.4c10532 (PMC11450719; doi:10.1021/acsami.4c10532)
Supplement: Supplementary file 1 — am4c10532_si_001.docx [file am4c10532_si_001.docx]

**Supporting information**

Phosphoramide Hydrogels as Biodegradable Matrices for Inkjet Printing and their Nano-Hydroxyapatite Composites

Mahsa Mostofizadeh^1,2^, Michael Kainz^3^*, Farzaneh Alihosseini^1^*, Stephan Haudum^2^, Mostafa Youssefi^1^, Peter Bauer^3^, Iurii Gnatiuk^4^, Oliver Brüggemann^2^, Katja Zembsch^5^, Uwe Rinner^5^, Catarina Coelho^6^, Elena Guillén^3^ and Ian Teasdale^2^*

^1^Department of Textile Engineering, Isfahan University of Technology, Isfahan 84156-83111, Iran.

^2^Institute of Polymer Chemistry, Johannes Kepler University, 4040 Linz, Austria.

^3^Functional Surfaces and Nanostructures, Profactor GmbH, 4407 Steyr-Gleink, Austria.

^4^Tiger Coatings GmbH & Co. KG, TIGER Inks, 4600 Wels, Austria.

^5^Institute of Applied Chemistry, IMC University of Applied Sciences Krems, Piaristengasse 1, 3500 Krems, Austria

^6^FLUIDINOVA, S.A., 4475-188 Maia, Portugal.

* Ian Teasdale − Institute of Polymer Chemistry, Johannes Kepler University Linz, 4040 Linz, Austria; Email: ian.teasdale@jku.at

* Farzaneh Alihosseini − Department of Textile Engineering, Isfahan University of Technology, Isfahan 84156-83111, Iran; Email: fhosseini@iut.ac.ir

* Michael Kainz - Functional Surfaces and Nanostructures, Profactor GmbH, 4407 Steyr-Gleink, Austria; Email: michael.kainz@profactor.at


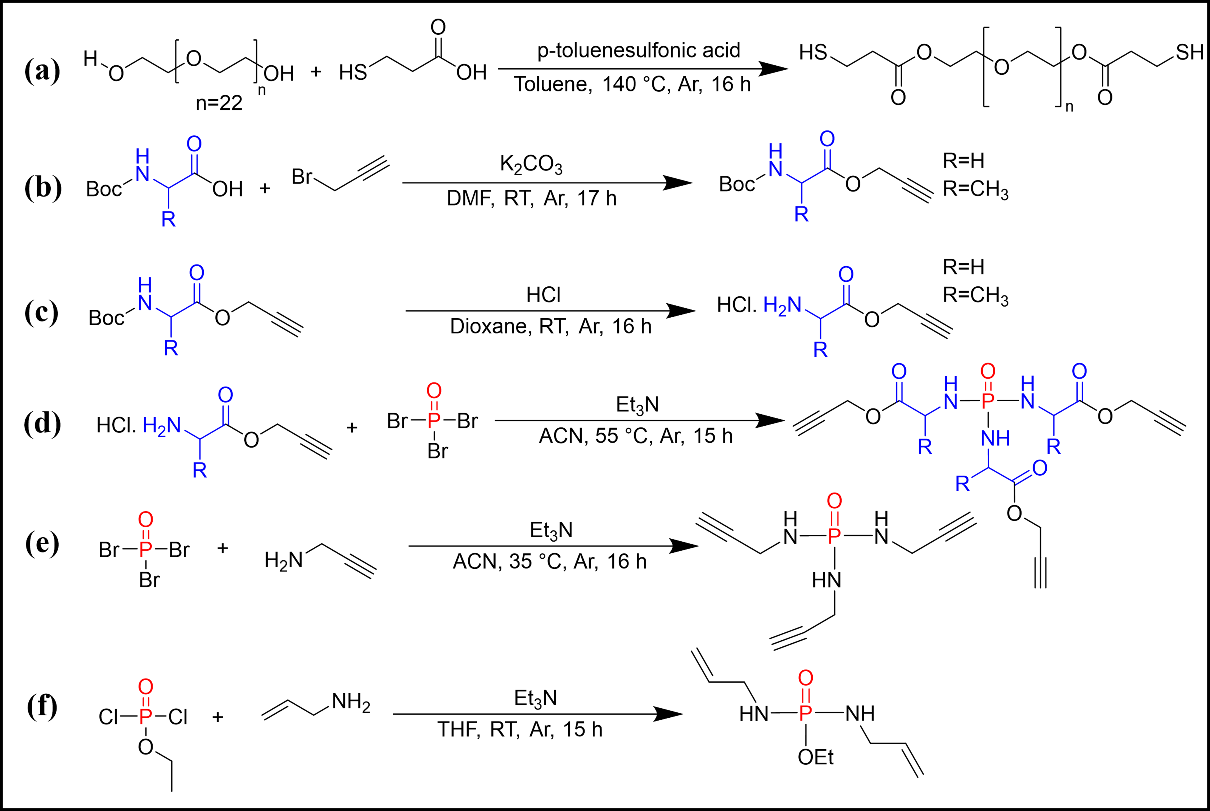


**Scheme S1.** Chemical schemes of the substituents and monomers.

***
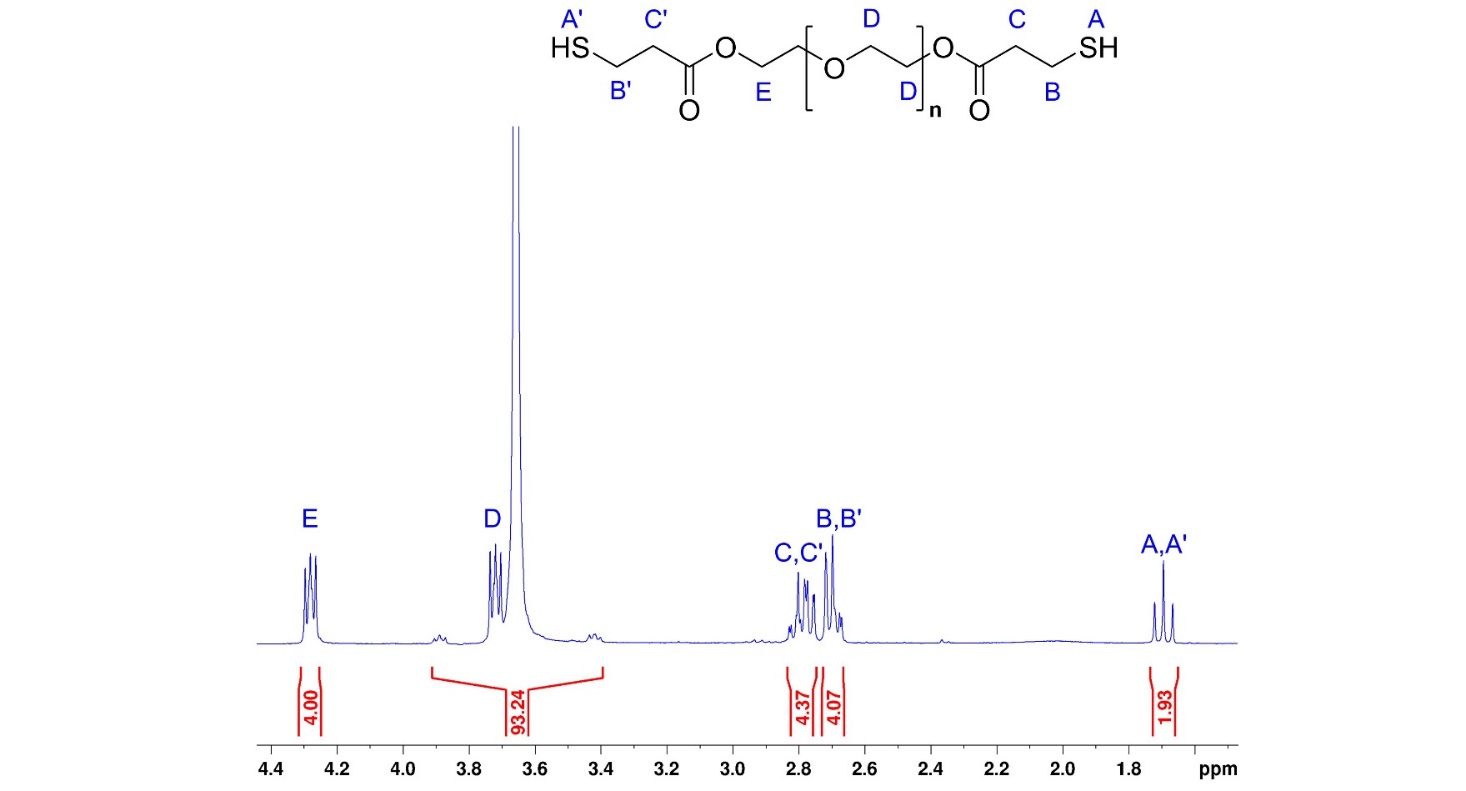
***

**Figure S1.** ^1^H NMR spectrum of PEG(SH)_2_ inclusive of integrals and proton assignments (300 MHz, CDCl_3_).

*
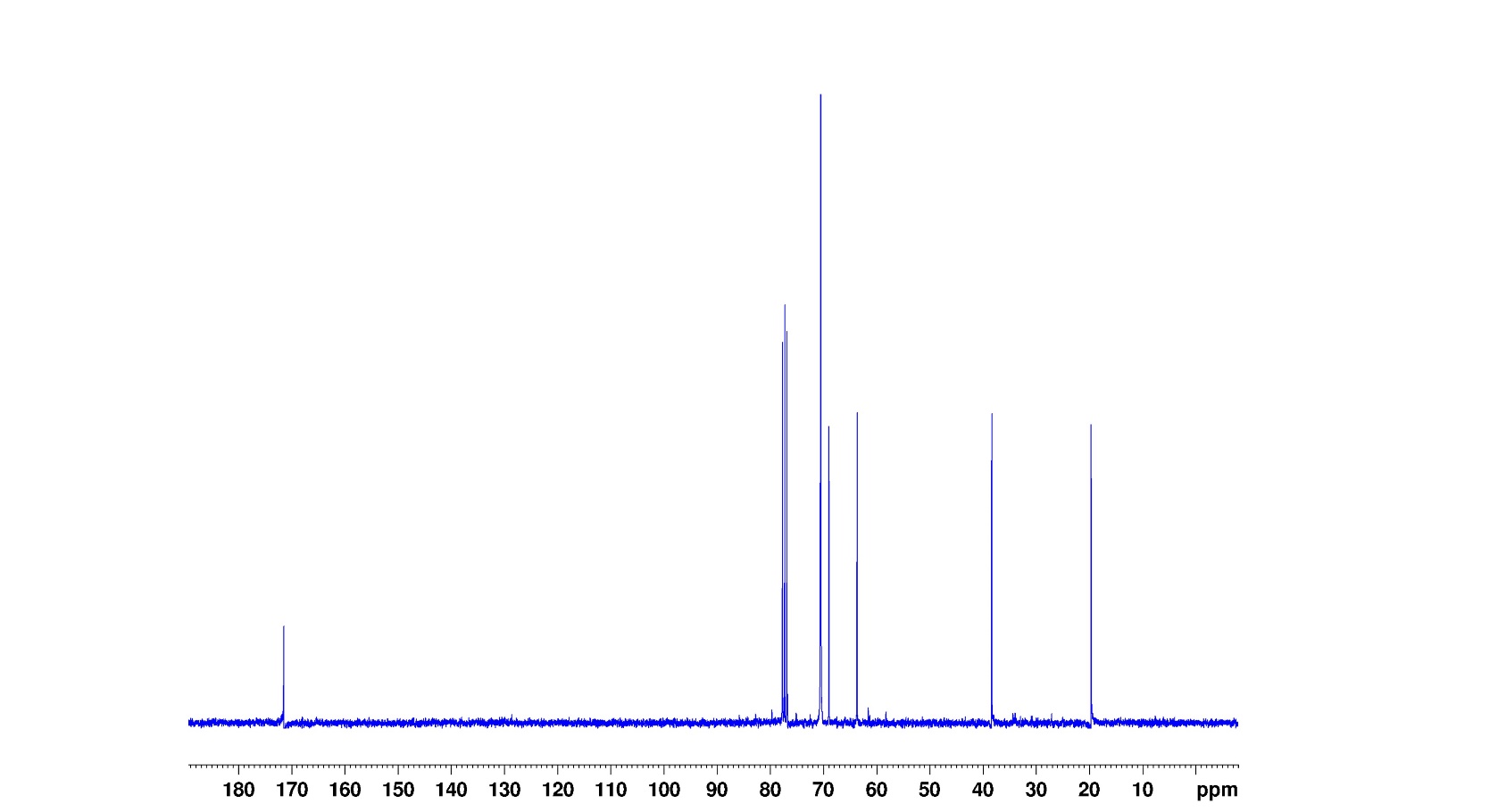
*

**Figure S2.** ^13^C NMR spectrum of PEG(SH)_2_ (75 MHz, CDCl_3_).

*
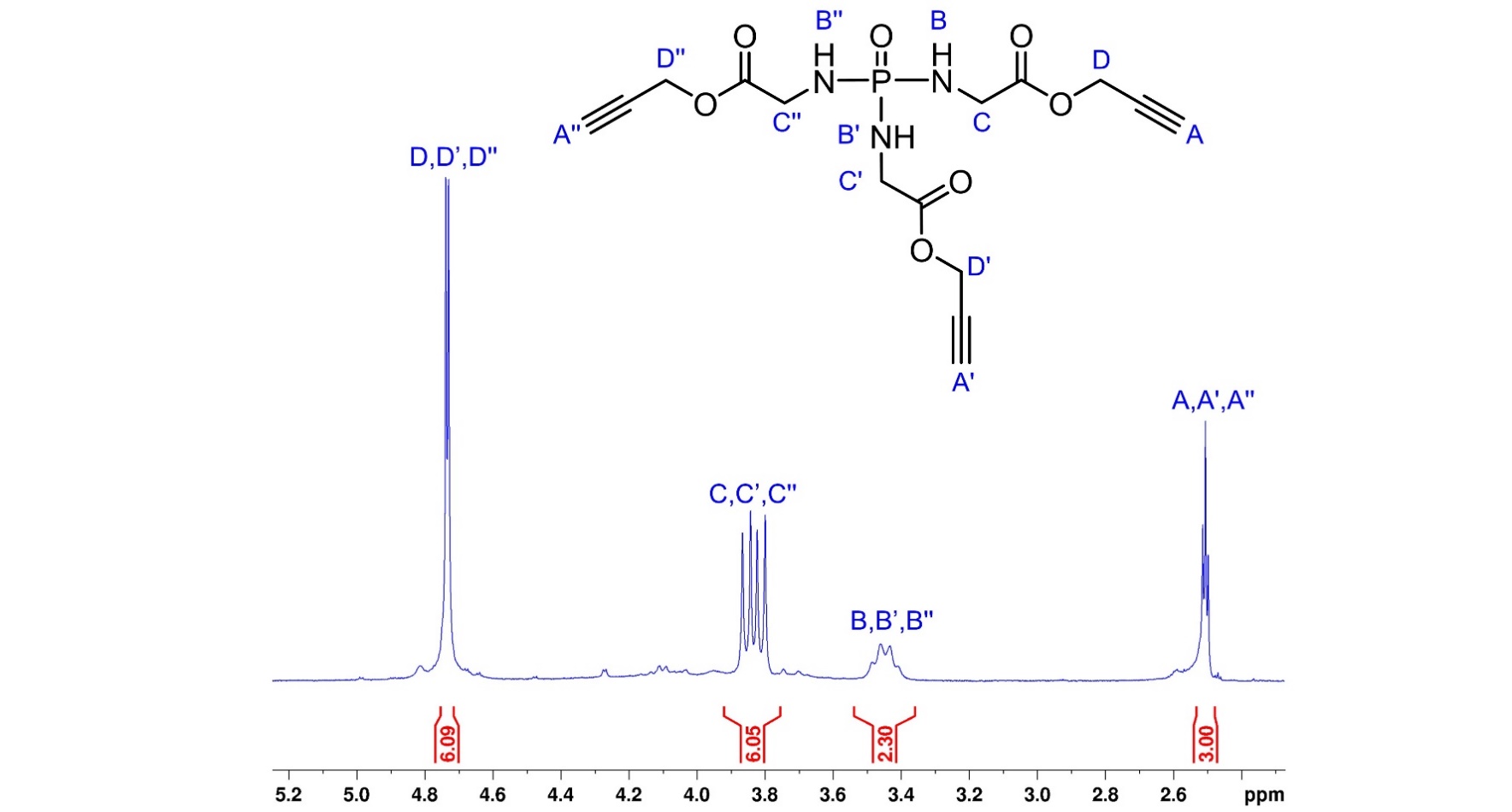
*

**Figure S3.** ^1^H NMR spectrum of Gly-APA inclusive of integrals and proton assignments (300 MHz, CDCl_3_).

*
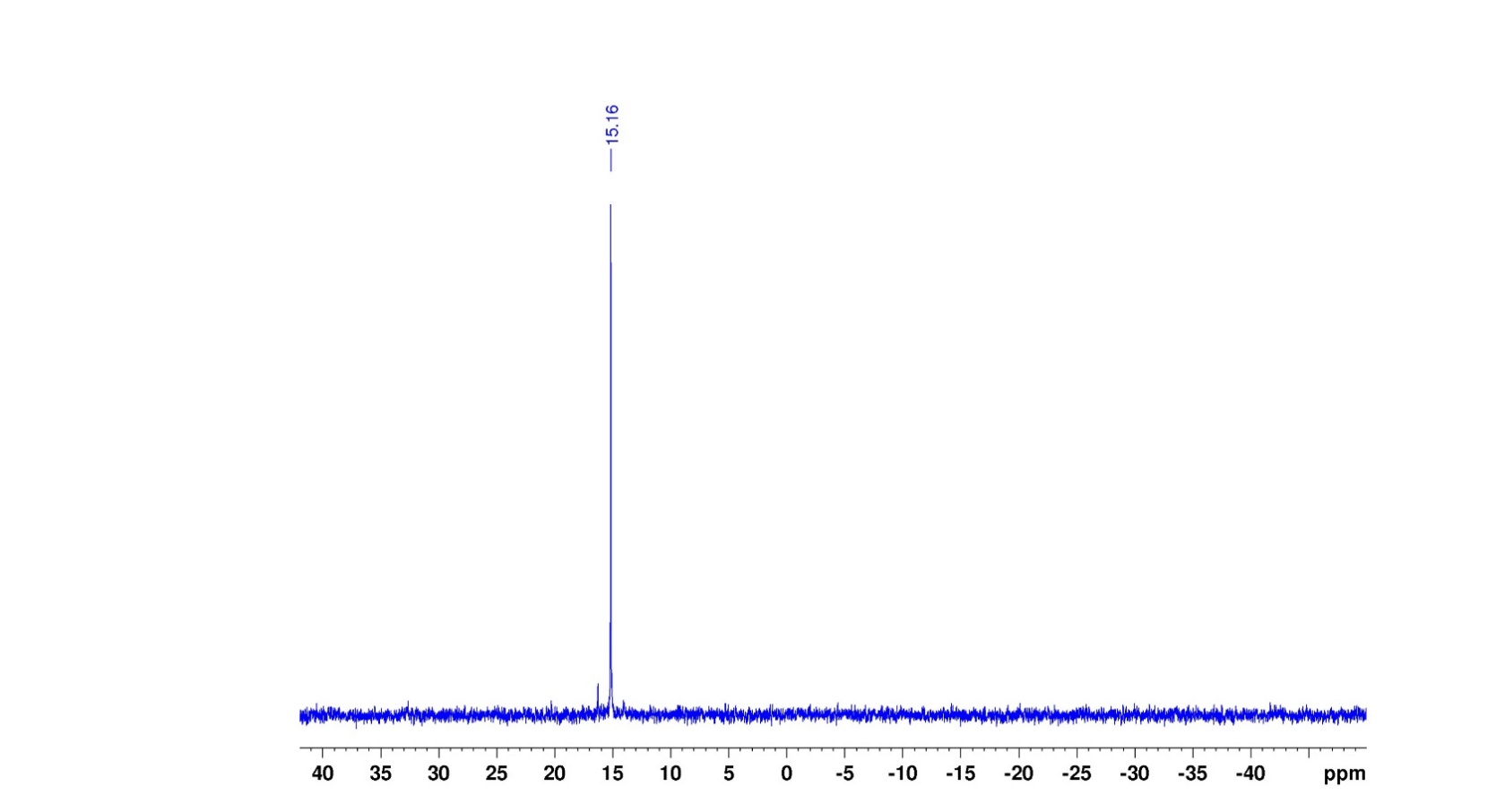
*

**Figure S4**. ^31^P NMR spectrum of Gly-APA (121 MHz, CDCl_3_).

*
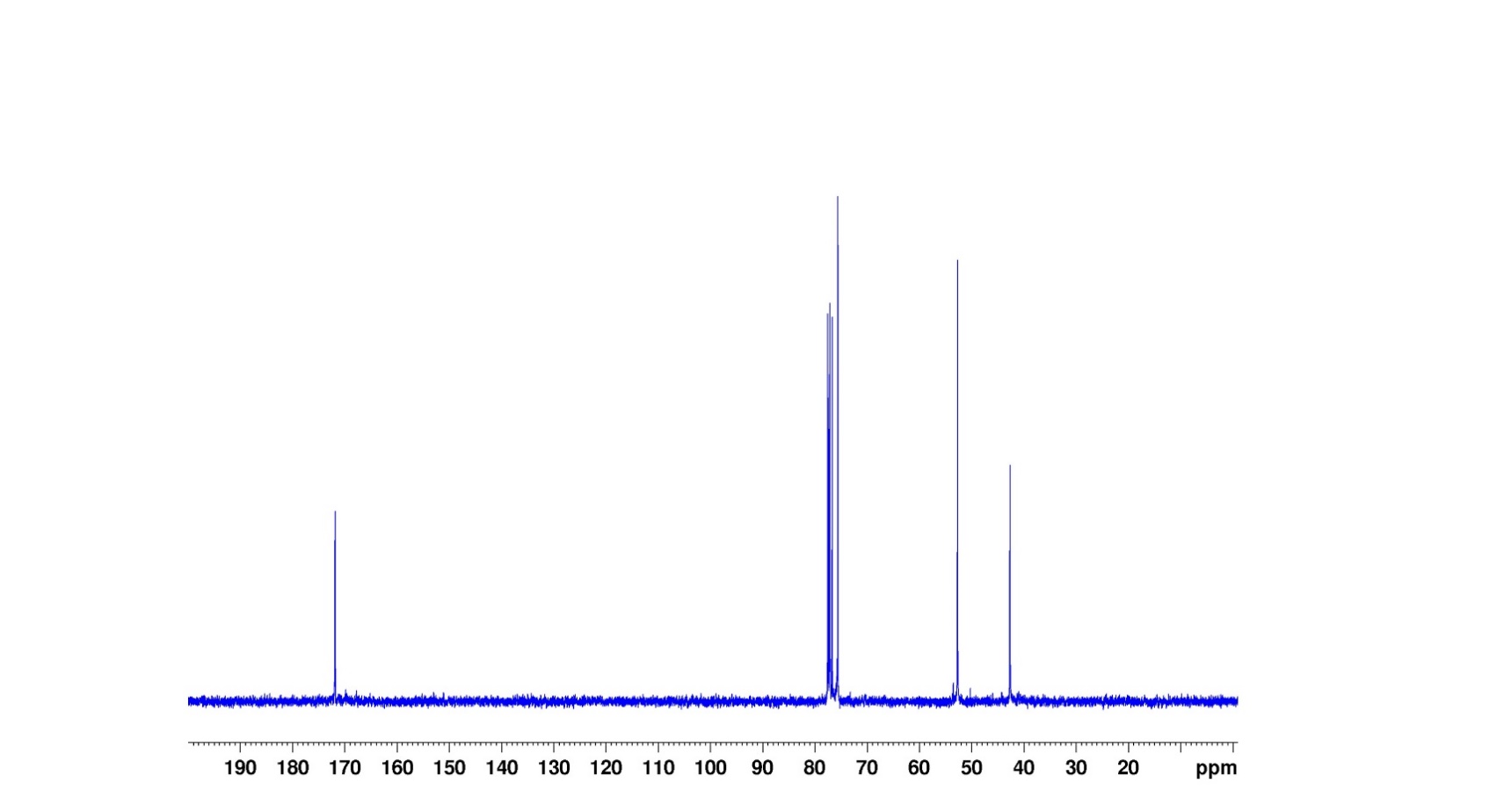
*

**Figure S5.** ^13^C NMR spectrum of Gly-APA (75 MHz, CDCl_3_).


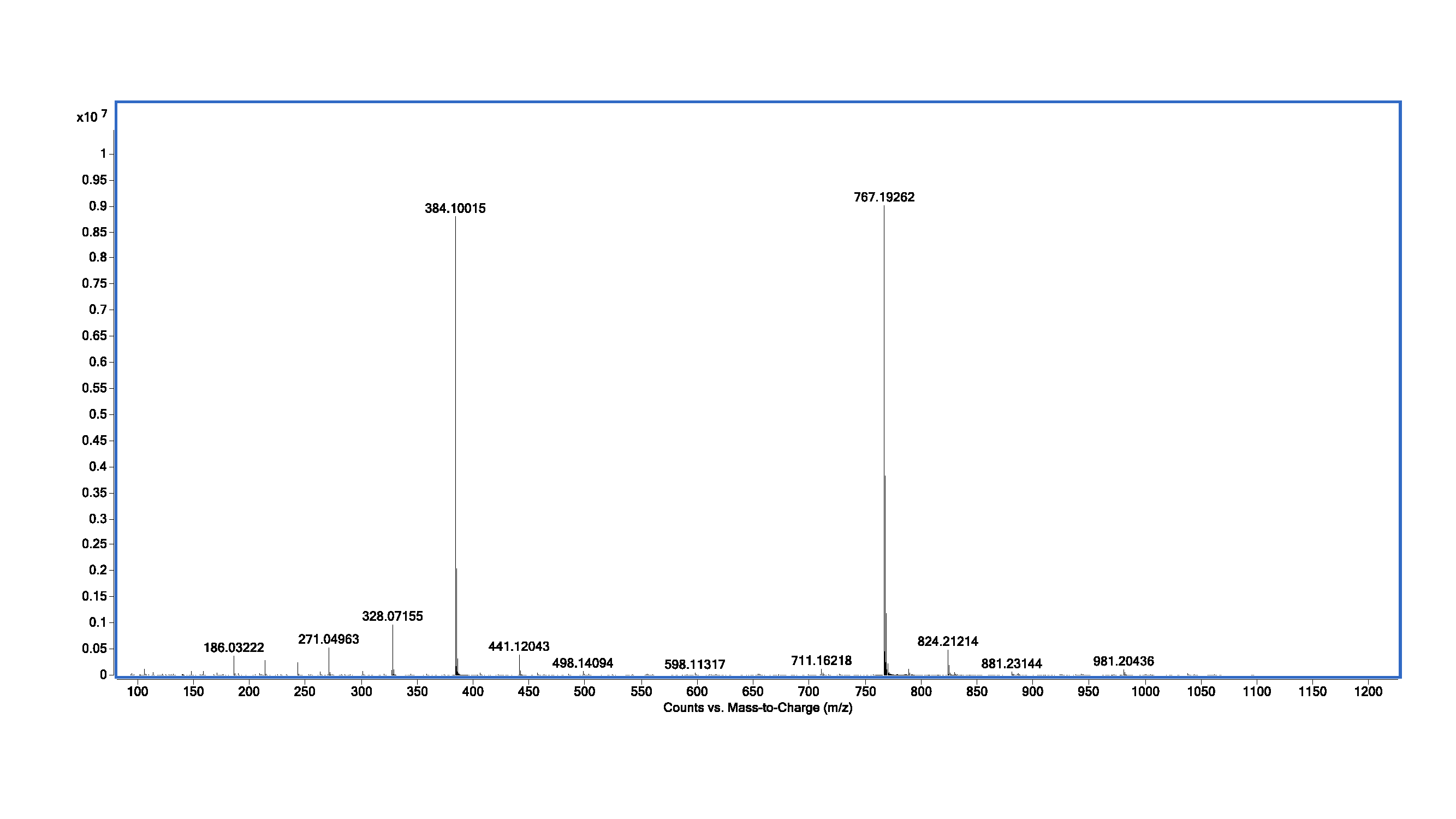


**Figure S6**. HR-ESI-MS spectrum of Gly-APA; m/z 384.1001 [M + H]^+^, 767.1926 [2M + H]^+^.

*
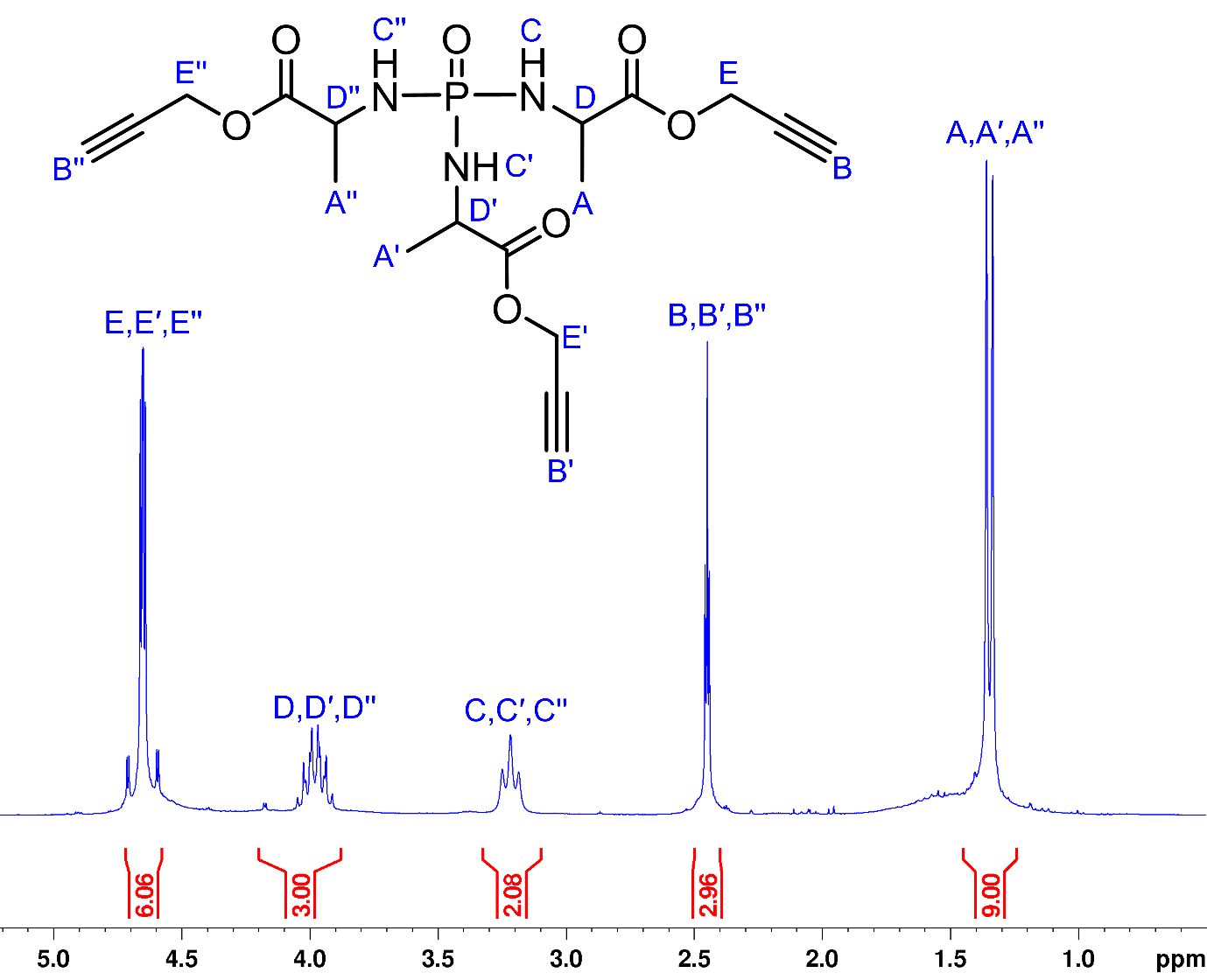
*

**Figure S7.** ^1^H NMR spectrum of Ala-APA inclusive of integrals and proton assignments (300 MHz, CDCl_3_).

*
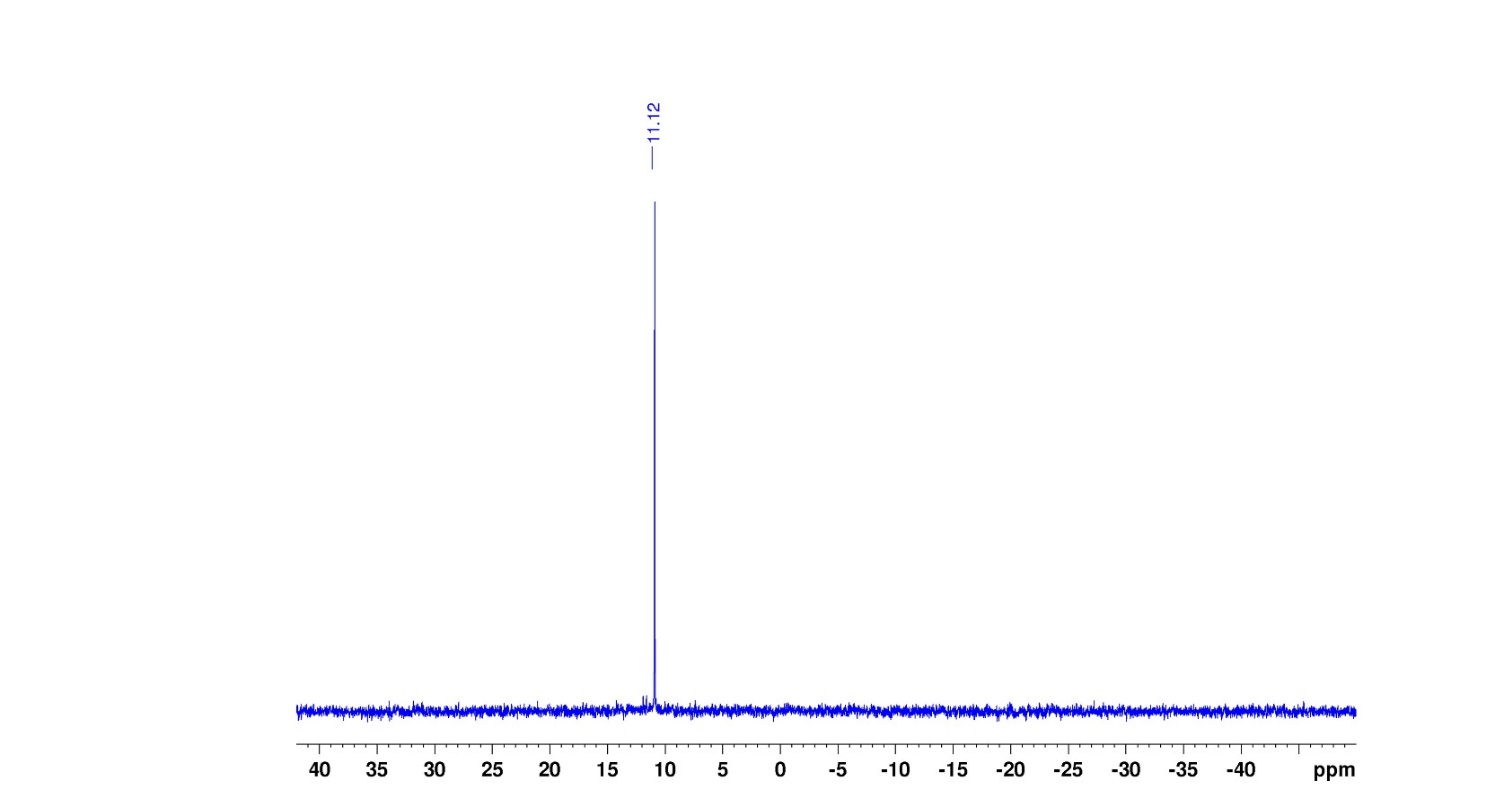
*

**Figure S8.** ^31^P NMR spectrum of Ala-APA (121 MHz, CDCl_3_).

*
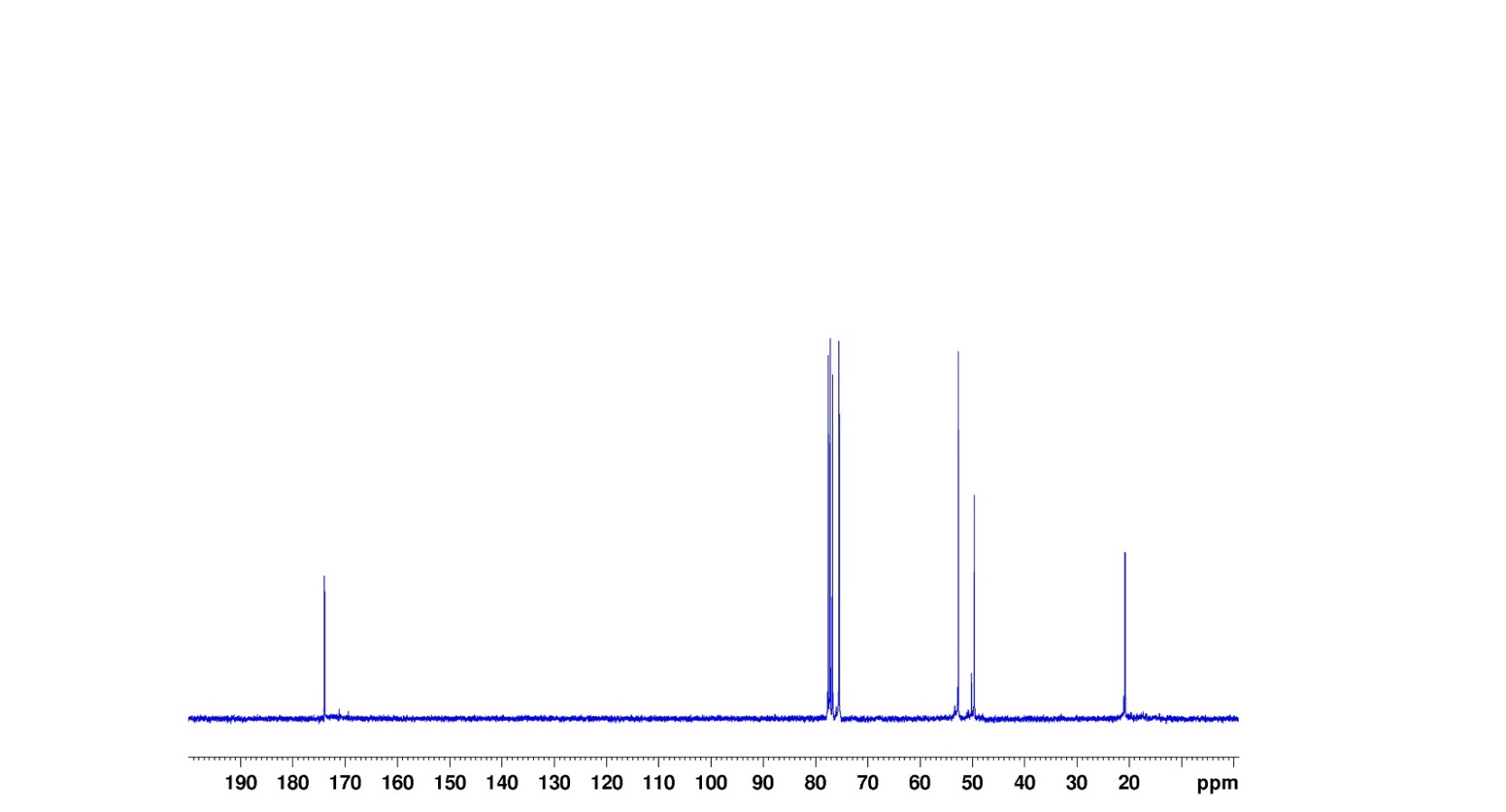
*

**Figure S9.** ^13^C NMR spectrum of Ala-APA (75 MHz, CDCl_3_).

*
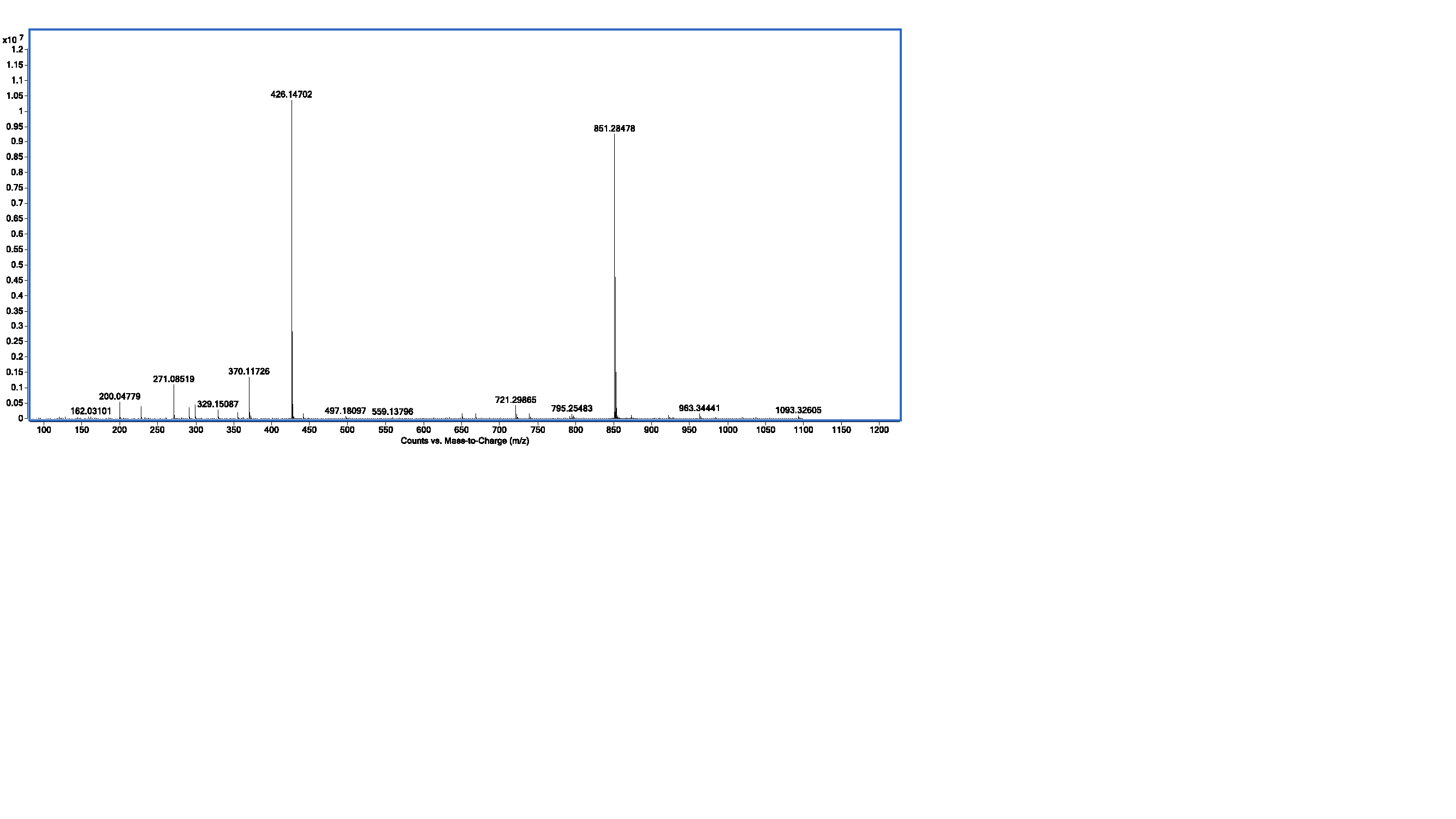
*

**Figure S10.** HR-ESI-MS spectrum of Ala-APA; m/z 426.1470 [M + H]^+^, 851.2848 [2M + H]^+^.

*
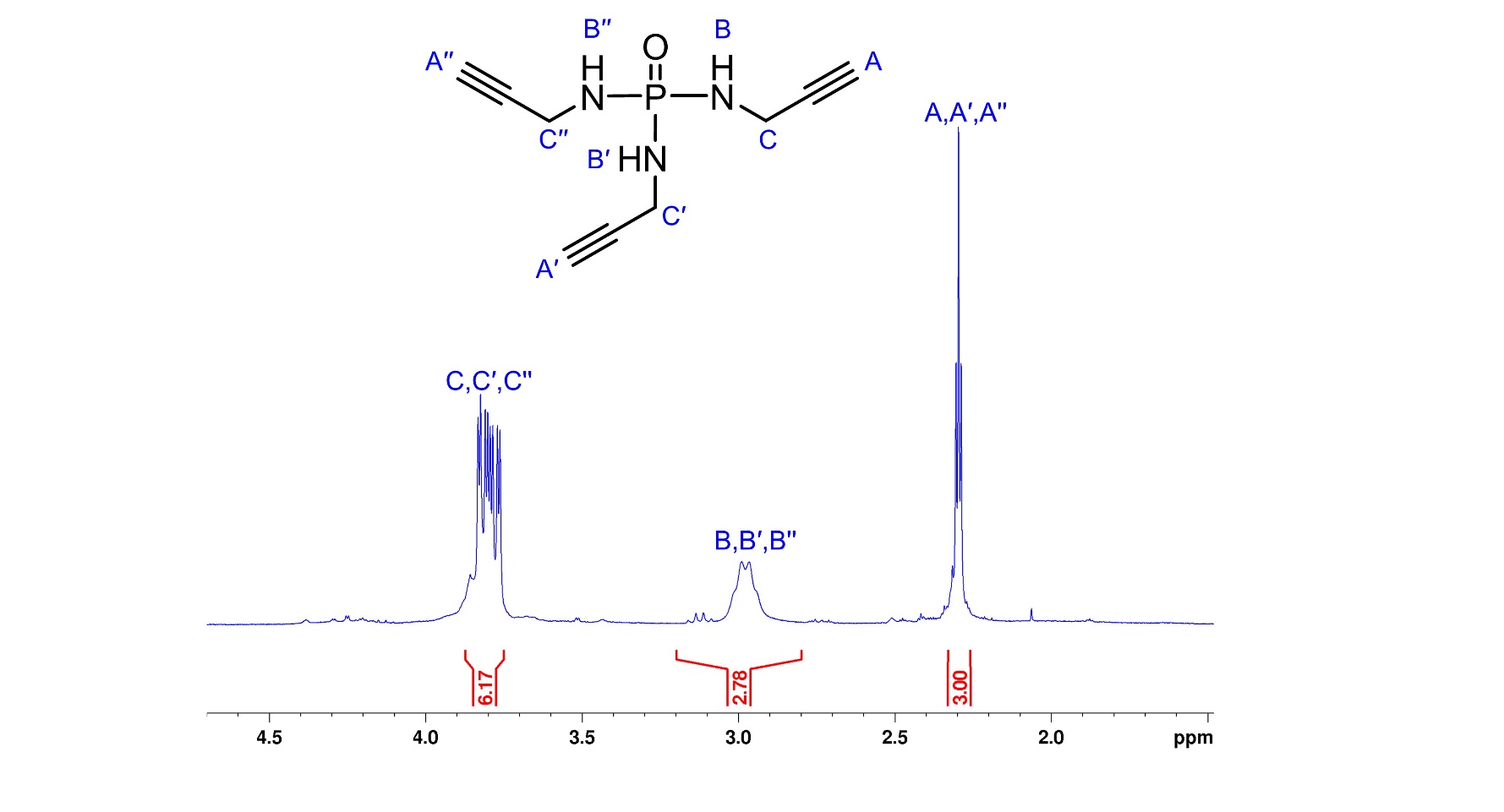
*

**Figure S11.** ^1^H NMR spectrum of Propargyl-PA inclusive of integrals and proton assignments (300 MHz, CDCl_3_).
**
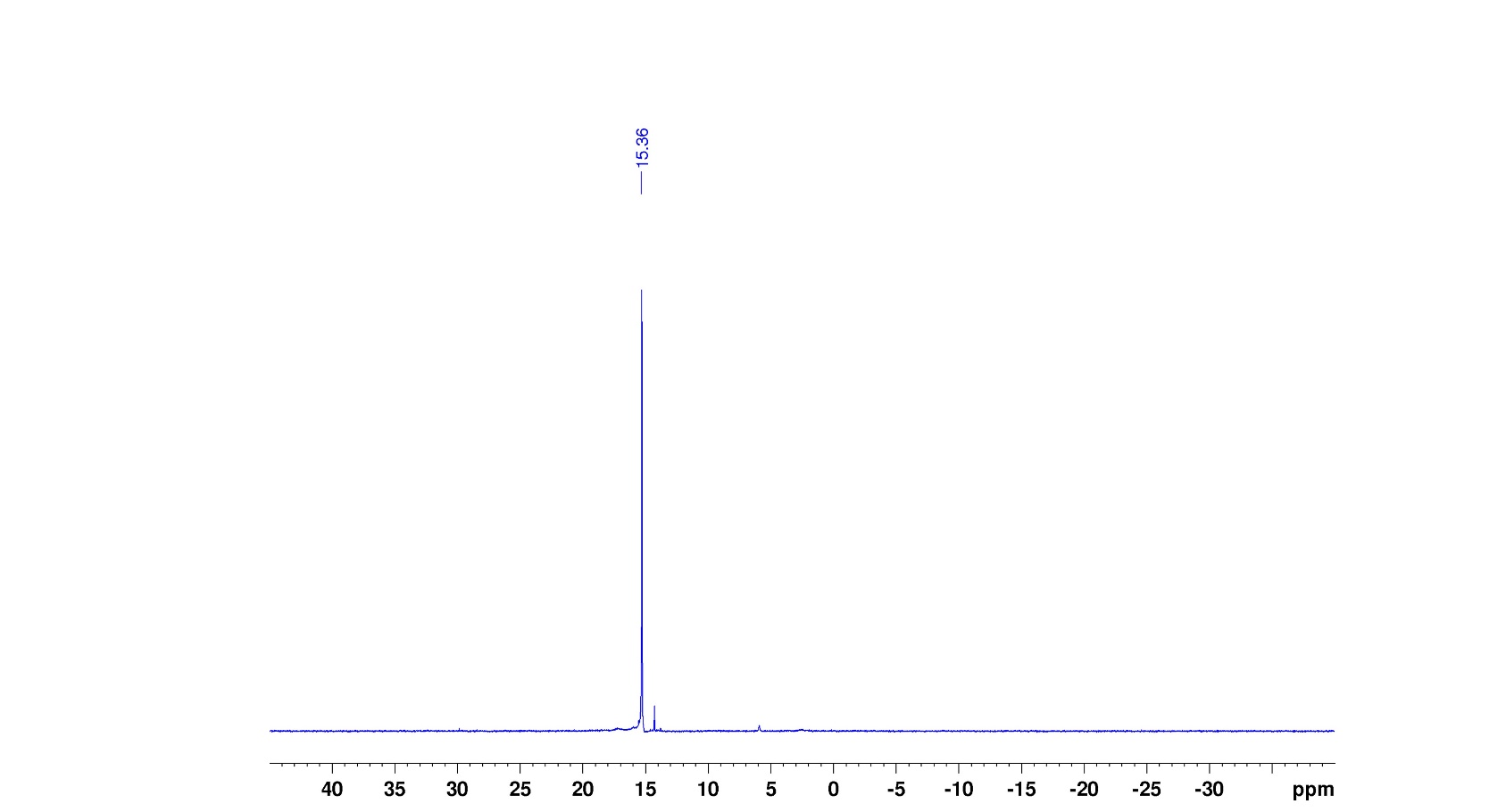
**

**Figure S12.** ^31^P NMR spectrum of Propargyl-PA (121 MHz, CDCl_3_).

*
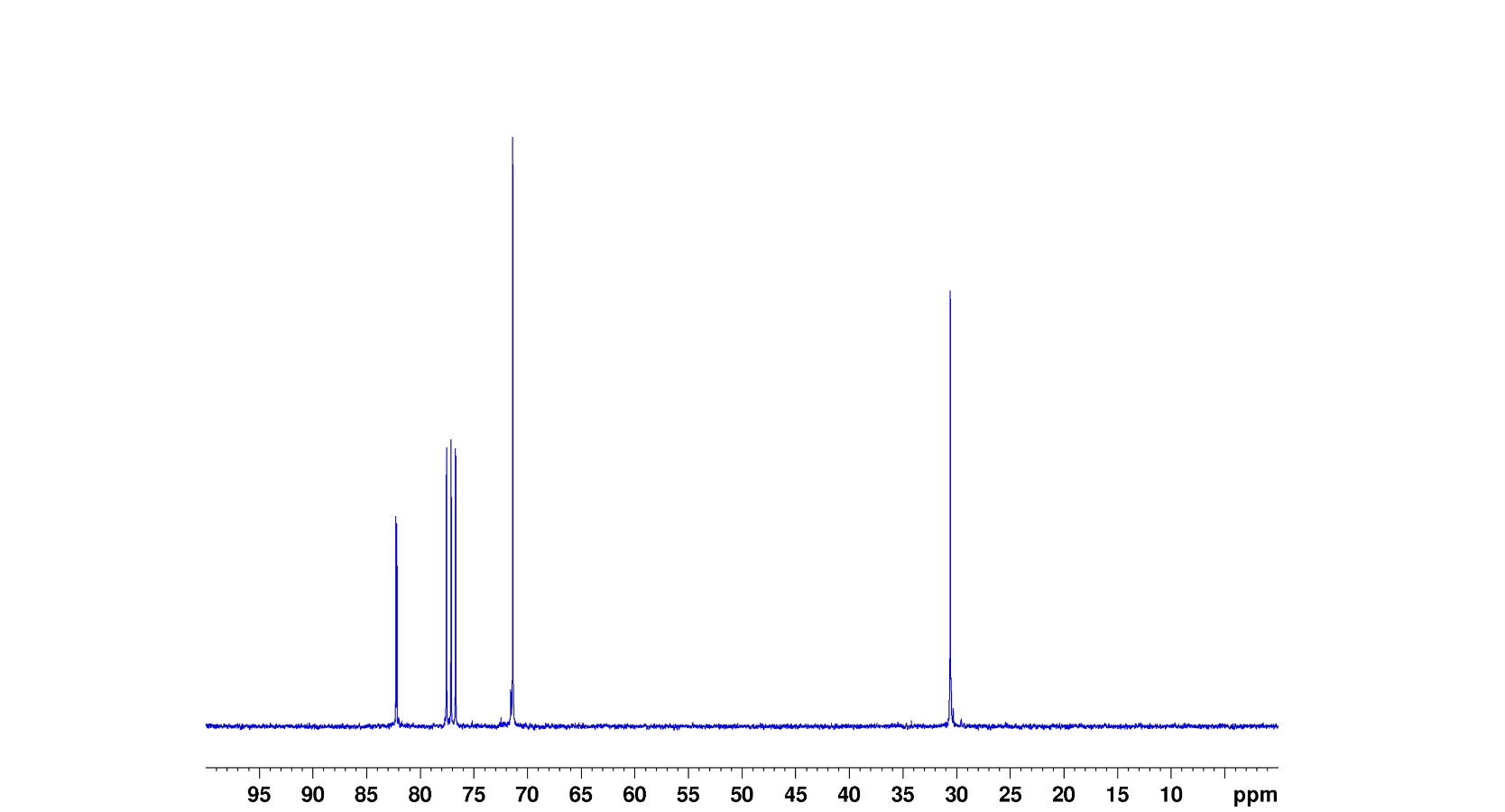
*

**Figure S13.** ^13^C NMR spectrum of Propargyl-PA (75 MHz, CDCl_3_).


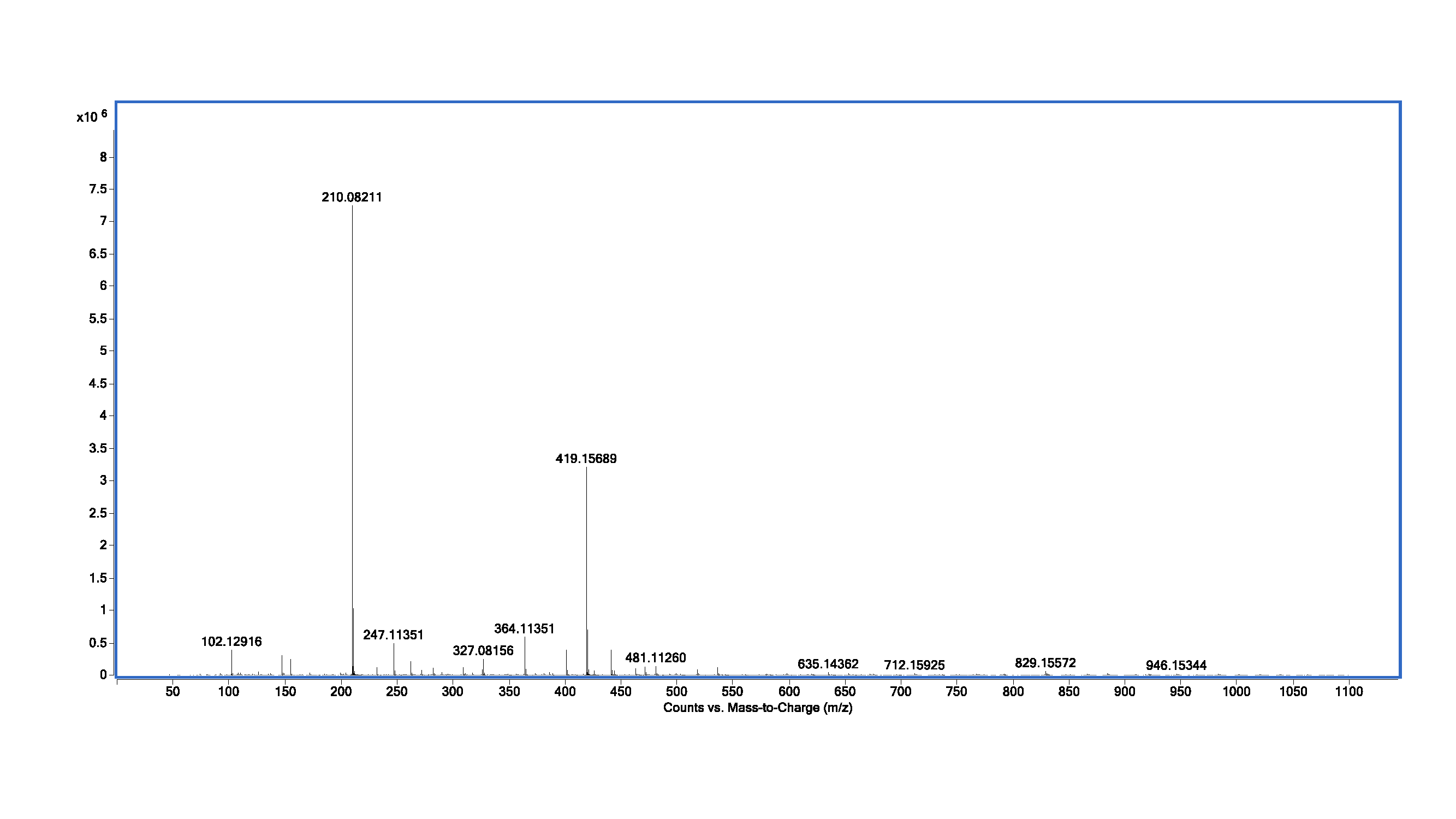


**Figure S14.** HR-ESI-MS spectrum of Propargyl-PA; m/z 210.0821 [M + H]^+^, 419.1569 [2M + H]^+^.

*
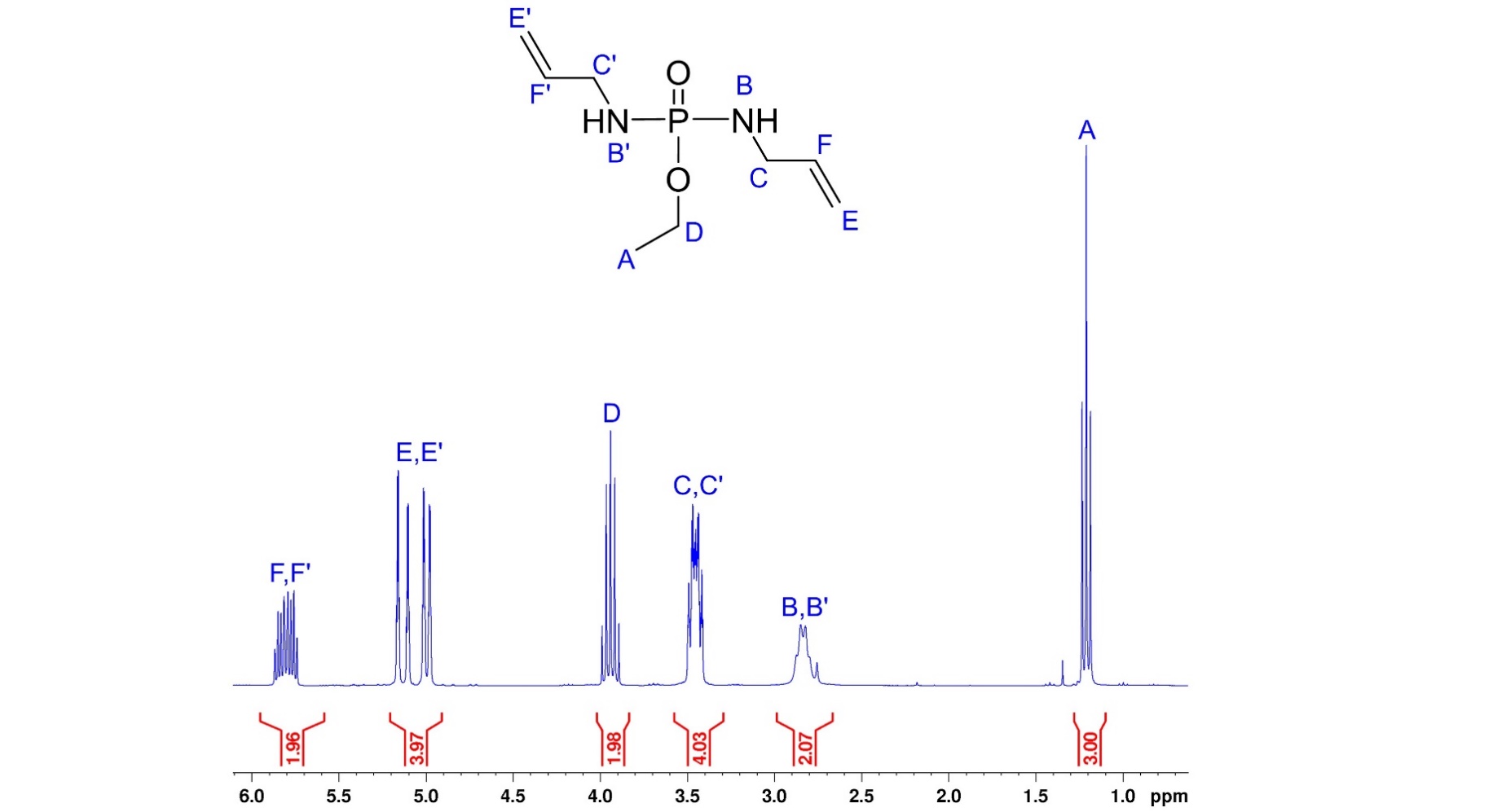
*

**Figure S15.** ^1^H NMR spectrum of Allyl-PdA inclusive of integrals and proton assignments (300 MHz, CDCl_3_).

*
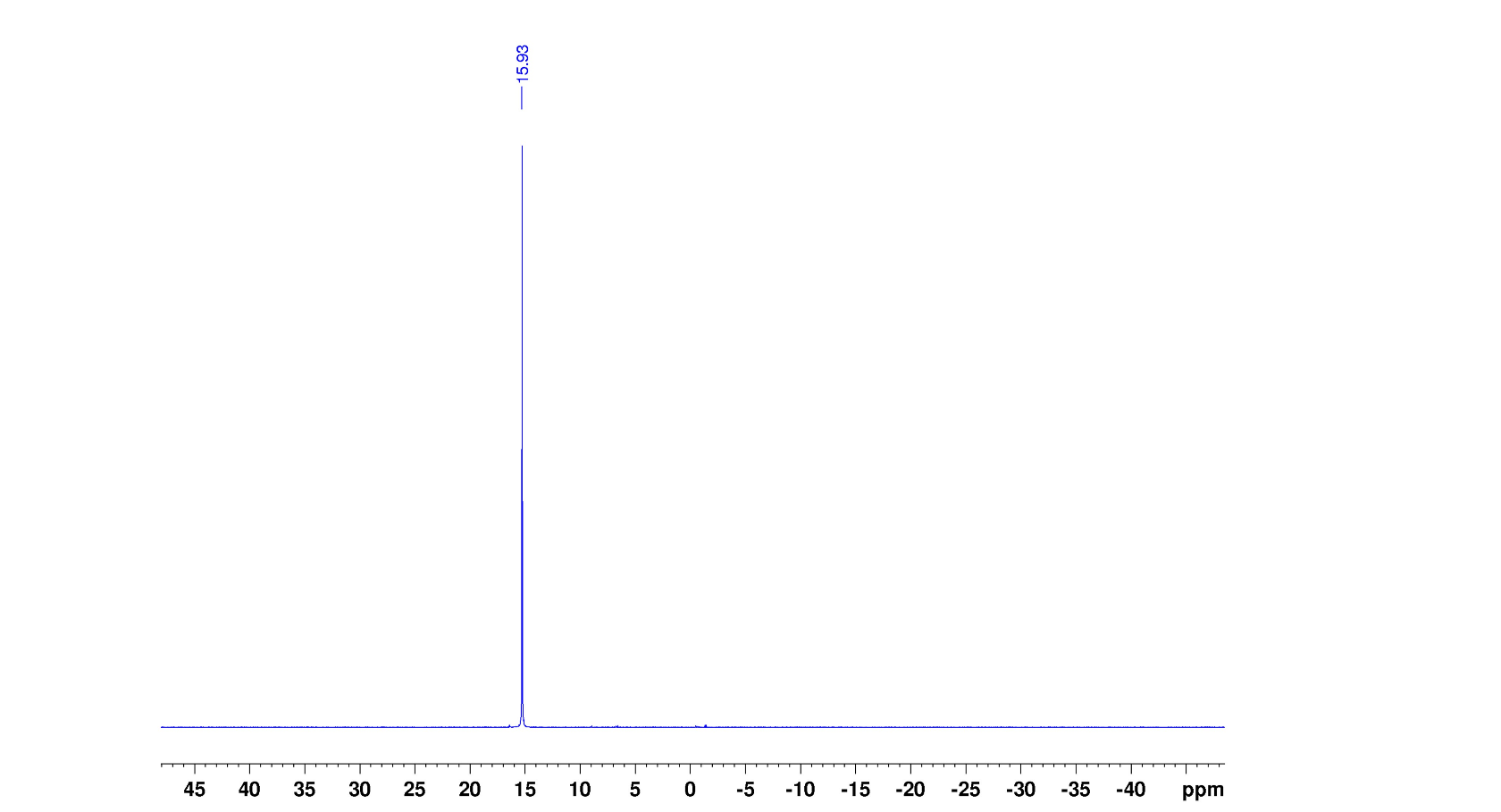
*

**Figure S16.** ^31^P NMR spectrum of Allyl-PdA (121 MHz, CDCl_3_).


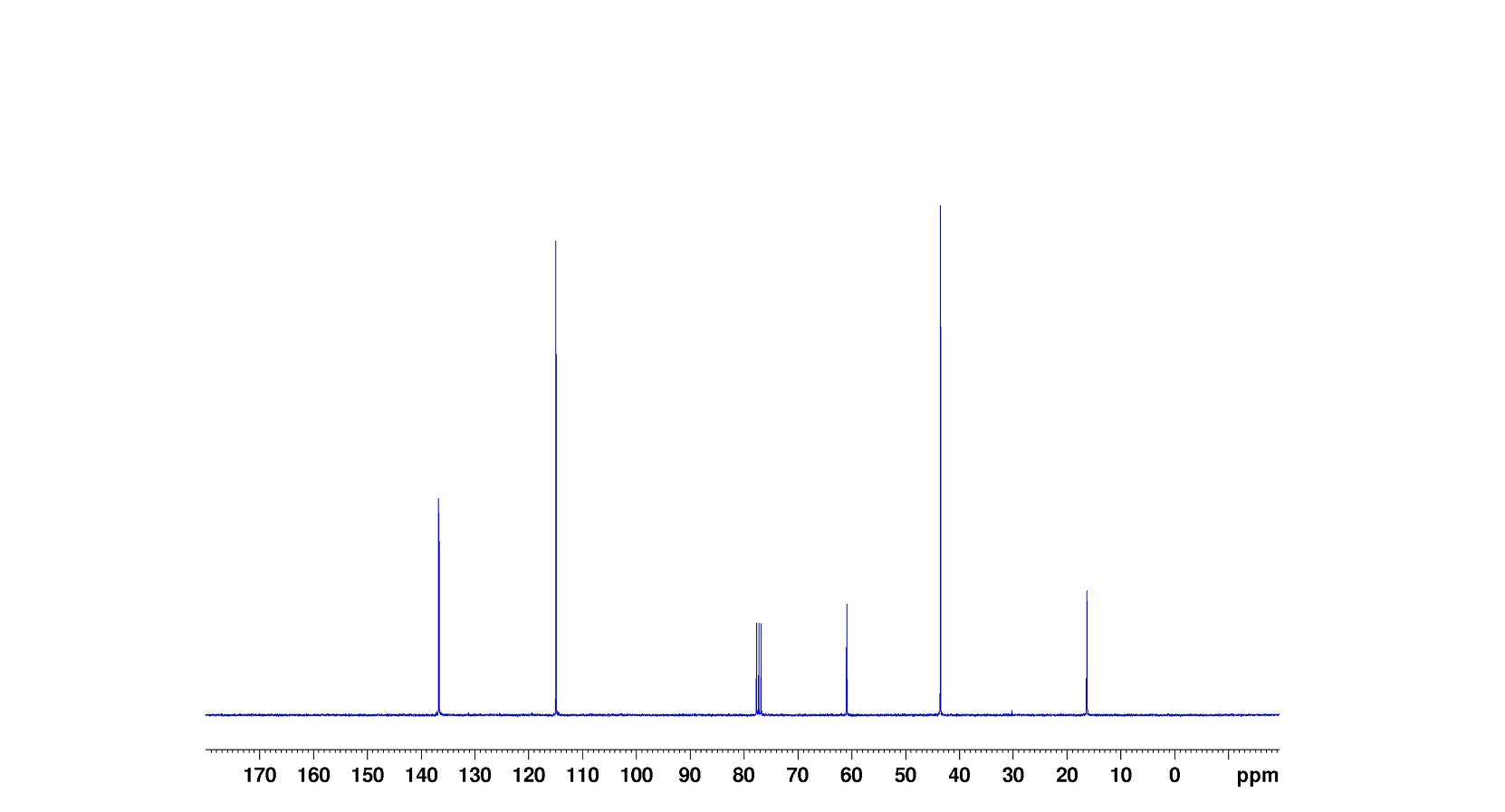


**Figure S17.** ^13^C NMR spectrum of Allyl-PdA (75 MHz, CDCl_3_).

*
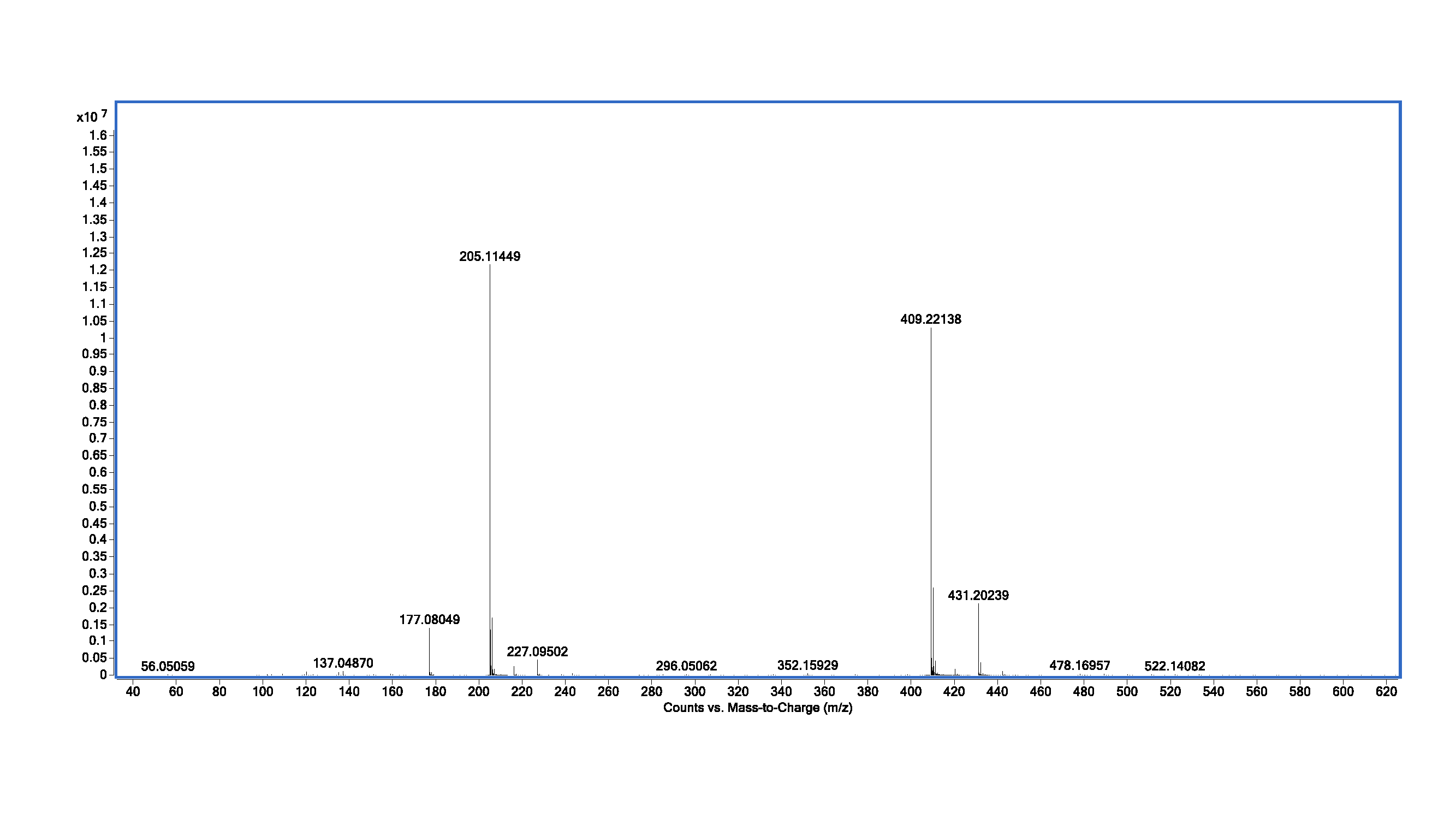
*

**Figure S18.** HR-ESI-MS spectrum of Allyl-PdA; m/z 205.1145 [M + H]^+^, 409.2214 [2M + H]^+^.


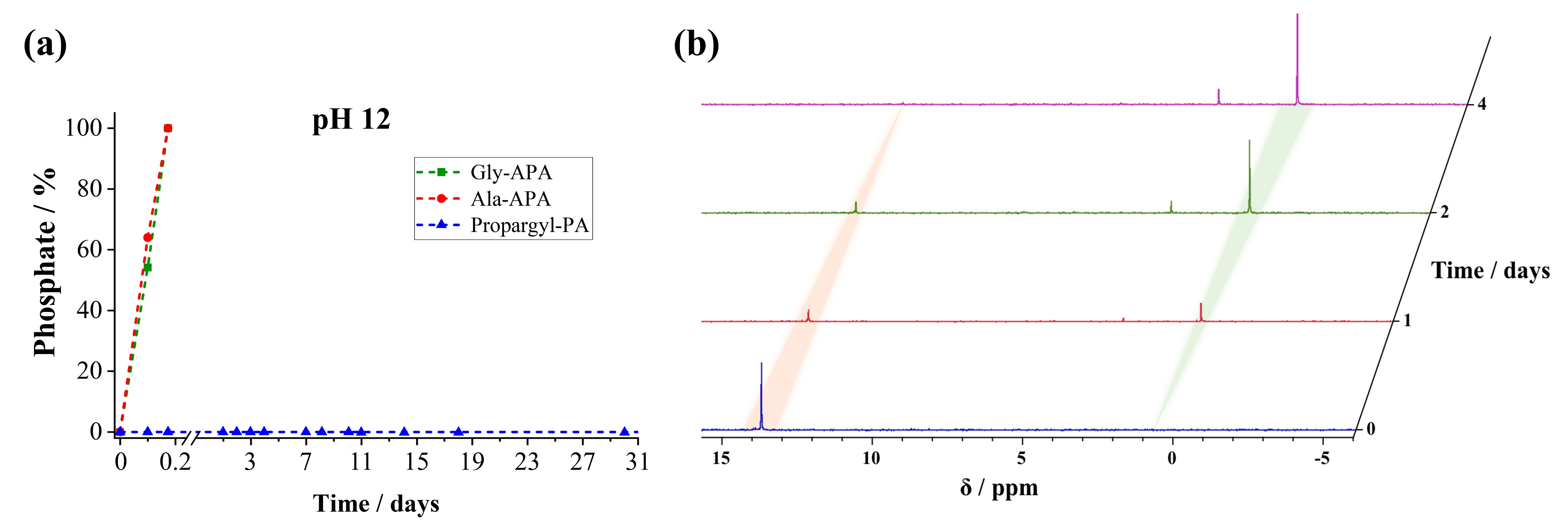


**Figure S19.** Hydrolysis of the synthesized monomers monitored by ^31^P NMR Spectroscopy: (a) Comparison of the hydrolysis of Gly-APA, Ala-APA, and Propargyl-PA monomers at 37 °C at pH 12. (b) Hydrolysis of Ala-APA monomer at pH 7.4. The signal of the monomer at 13.78 ppm decreases over time, while a new phosphate signal appears at 0.07 ppm. After 4 days, the monomer is completely degraded to the corresponding phosphate.


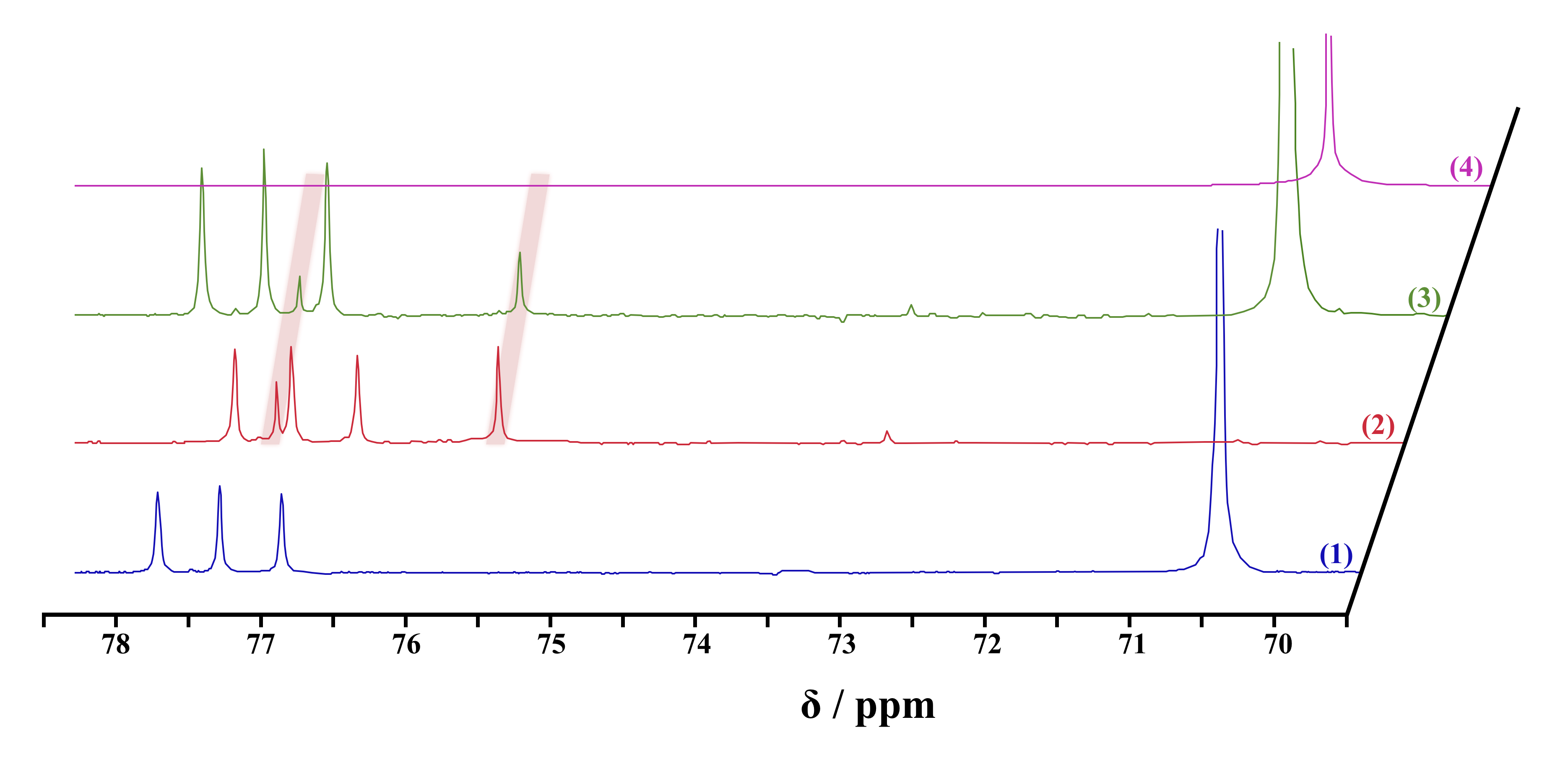


**Figure S20.** ^13^C NMR spectra of (1) PEG(SH)_2_ monomer, (2) Ala-APA monomer, (3) curing formulation of Ala-APA, PEG(SH)_2_ (4) cured Ala-APA hydrogel in a stacked view. During photopolymerization the triple bond signals of the Ala-APA monomer (75.6 and 77.2 ppm) visible in (2) and (3) disappear due to the thiol-yne click reaction with the thiol groups of PEG(SH)_2_. They are not visible anymore in the cured hydrogel (4), indicating a successful polymerization. (peaks at around 77.7, 77.3, and 76.8 are from CDCl_3_) ((1)-(3) 300 MHz, CDCl_3_; (4) 500 MHz).

*
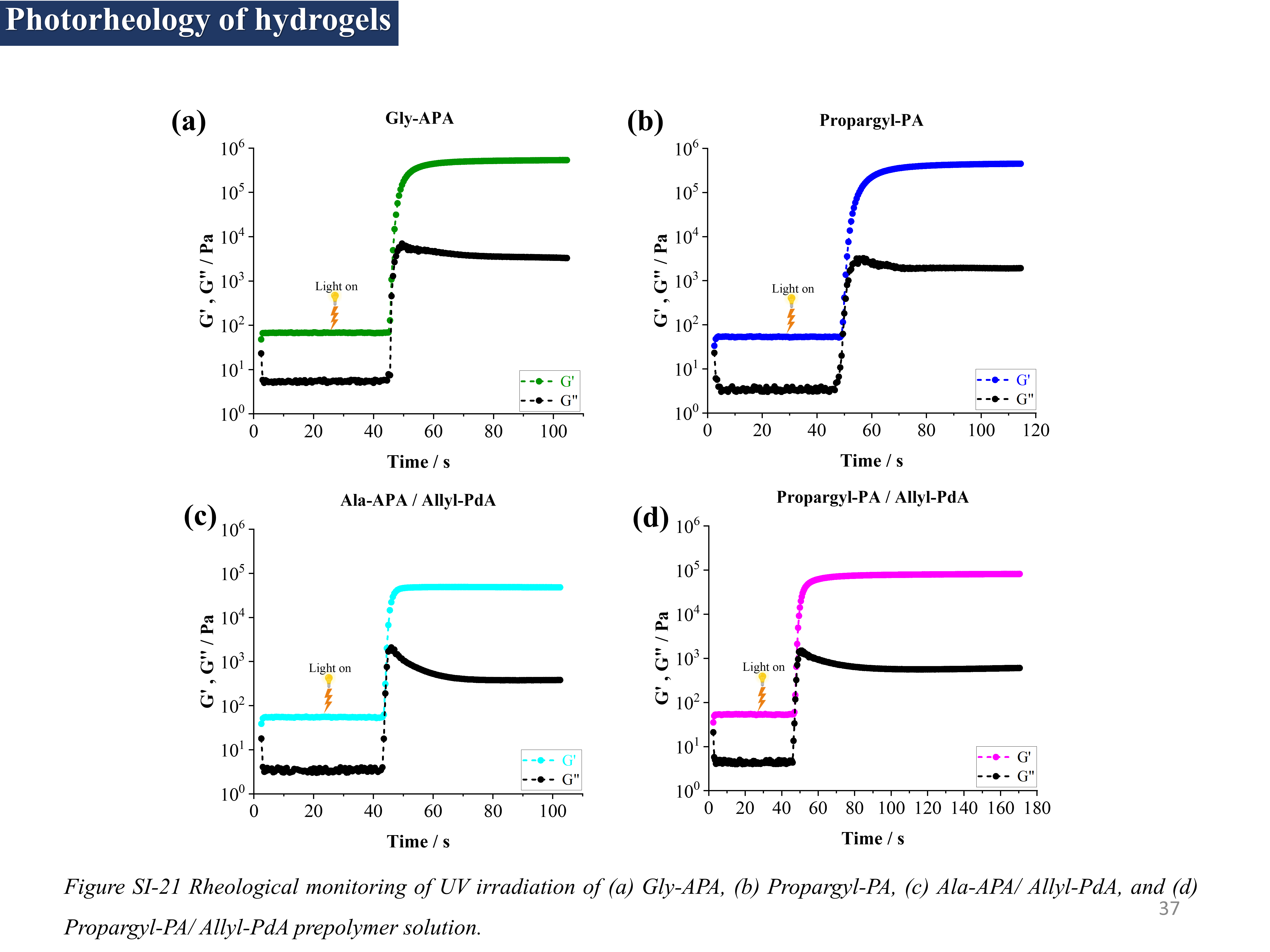
*

**Figure S21.** Rheological monitoring of UV irradiation of (a) Gly-APA, (b) Propargyl-PA, (c) Ala-APA/Allyl-PdA, and (d) Propargyl-PA/Allyl-PdA curing solution. All formulations exhibited a similar gelation time of 26.3 s.


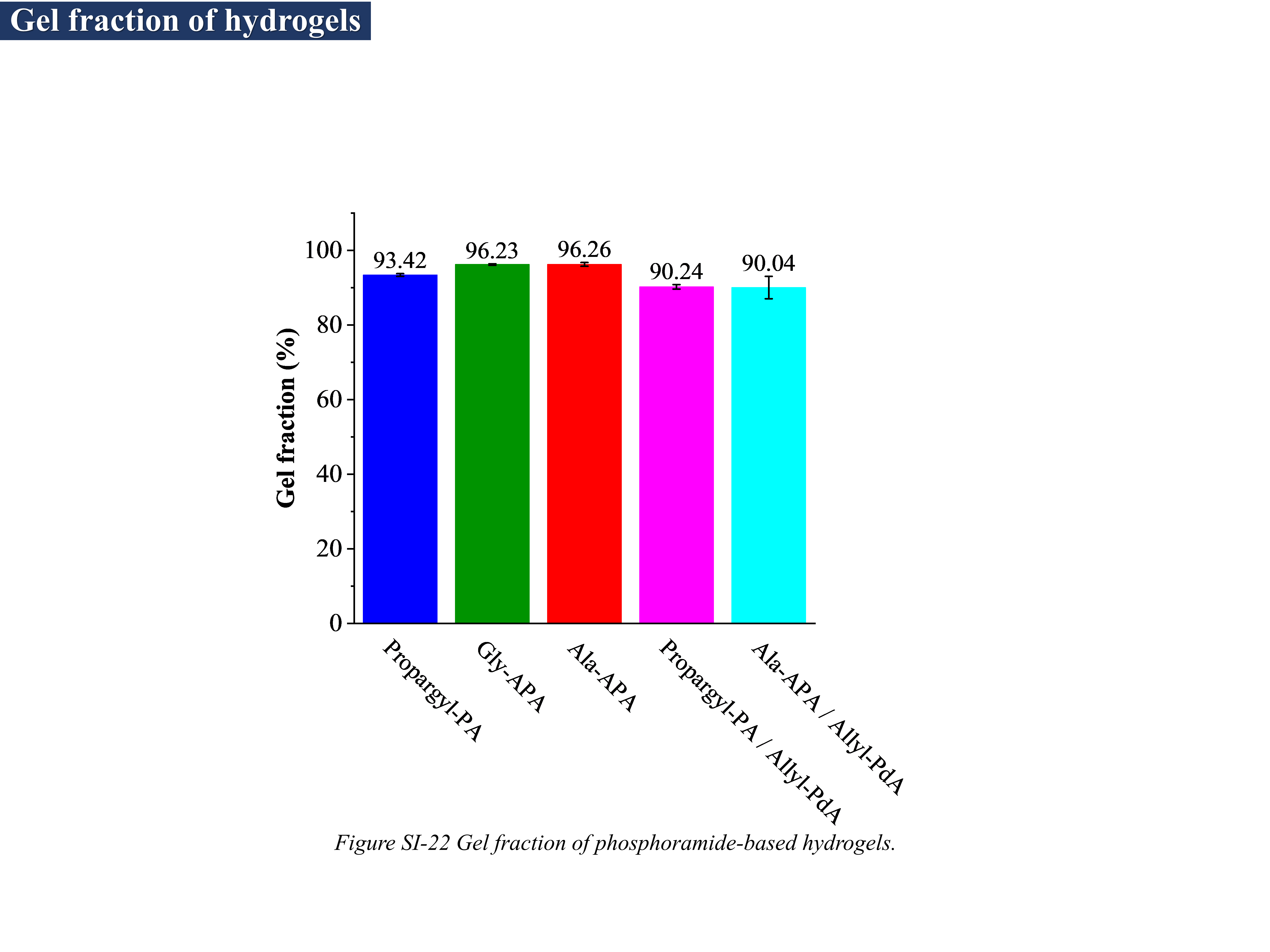


**Figure S22.** Gel fraction of phosphoramide-based hydrogels. All hydrogels showed good gel fraction above 90 % thereby indicating a low residual monomer content.

*
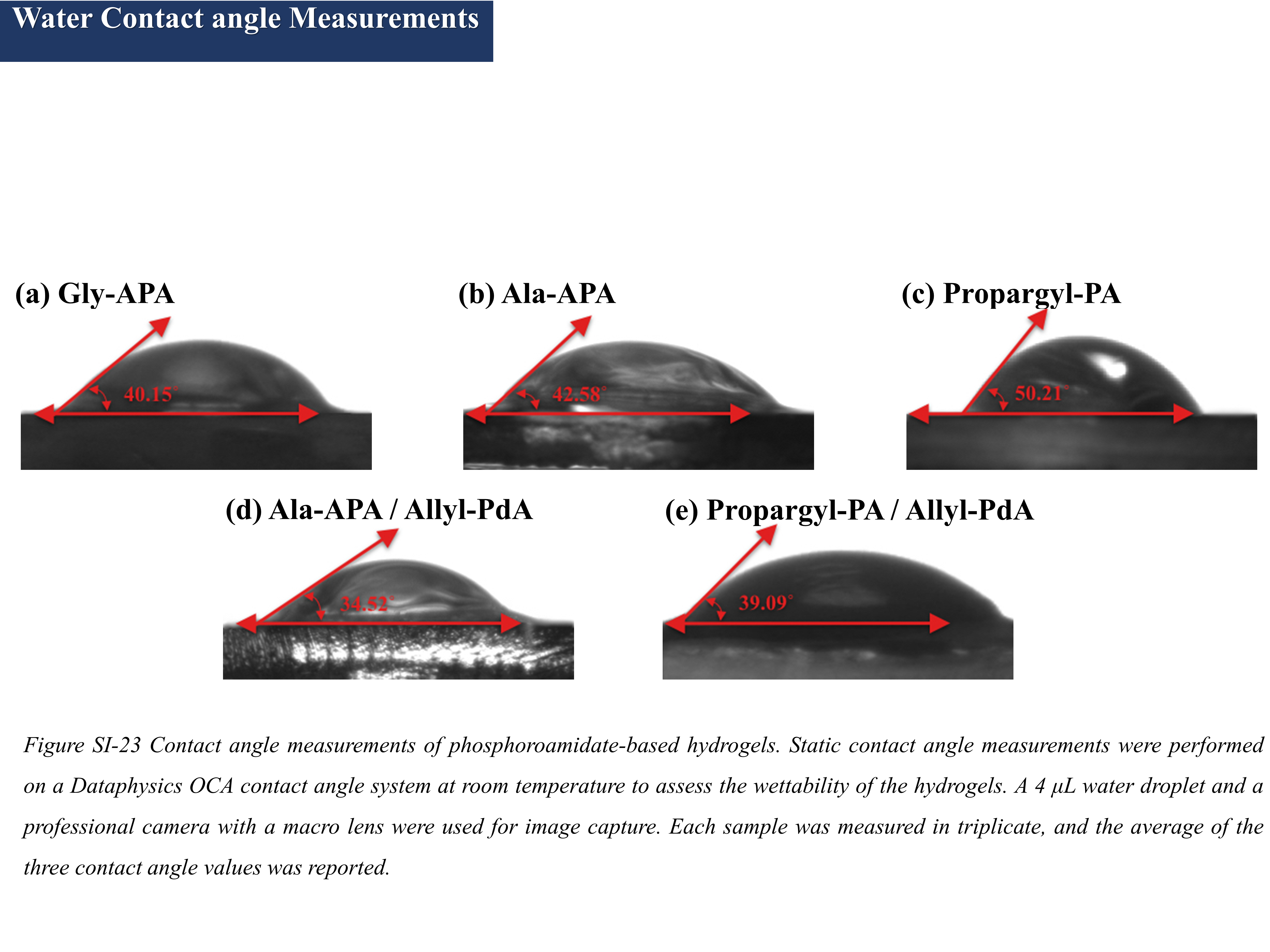
*

**Figure S23.** Contact angle measurements of phosphoramide-based hydrogels: (a) Gly-APA, (b) Ala-APA, (c) Propargyl-PA, (d) Ala-APA/Allyl-PdA, and (e) Propargyl-PA/Allyl-PdA. Static contact angle measurements were performed on a Dataphysics OCA contact angle system at RT to assess the wettability of the hydrogels. A 4 μL water droplet and a professional camera with a macro lens were used for image capture. Each sample was measured in triplicate, and the average of the three contact angle values was reported.


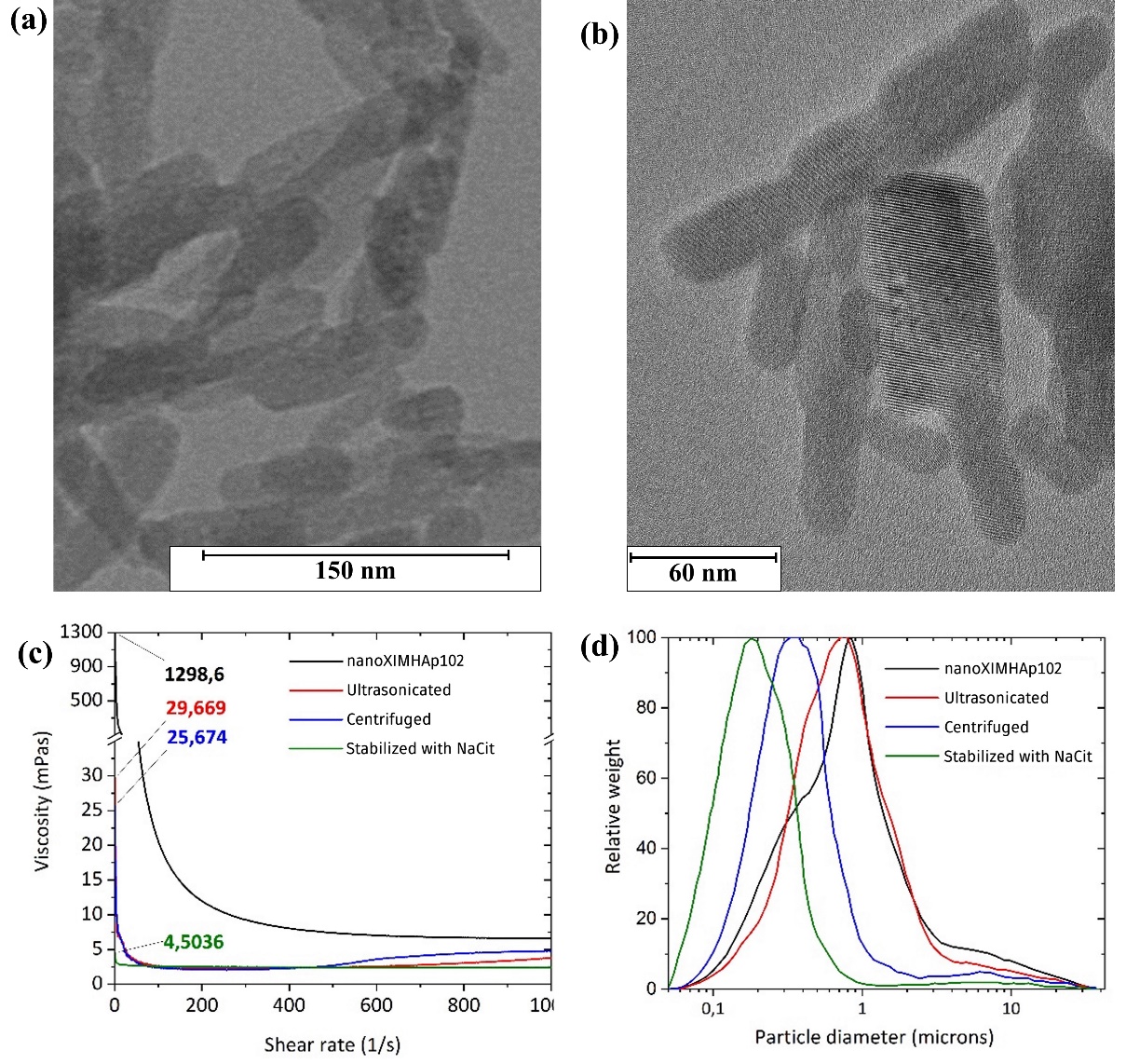


**Figure S24.** The TEM images of (a) nHAp and (b) its dispersion within the Propargyl-PA hydrogel composite (magnification 250000X). The influence of preparation procedure on ink properties: (c) Viscosity as a function of shear at 25 °C, (d) Particle size distribution.

Solid content, pH, polydispersity index (PDI), detected peaks and filtration properties using a 3 µm syringe glass fiber filter are summarized in Table S1. The solid content of nHAp according to the preparation procedure reduces from the initial 15 wt.% to 10.72 wt.%.

**Table S1.** Properties of the stabilized nHAp suspension after each preparation step.

| Preparation | HAp [wt.%] | pH | PDI | D50 Peaks [nm] | Filtration [g] |
| --- | --- | --- | --- | --- | --- |
| nanoXIM•HAp102 | 15 | 9 | 13.49 | 837 | - |
| Ultrasonicated | 15 | 9 | 9.77 | 747 | - |
| Centrifuged | 10.82 | 9.10 | 8.13 | 336 | - |
| Stabilized with sodium citrate | 10.72 | 11.12 | 6.86 | 181 | 18.50 |


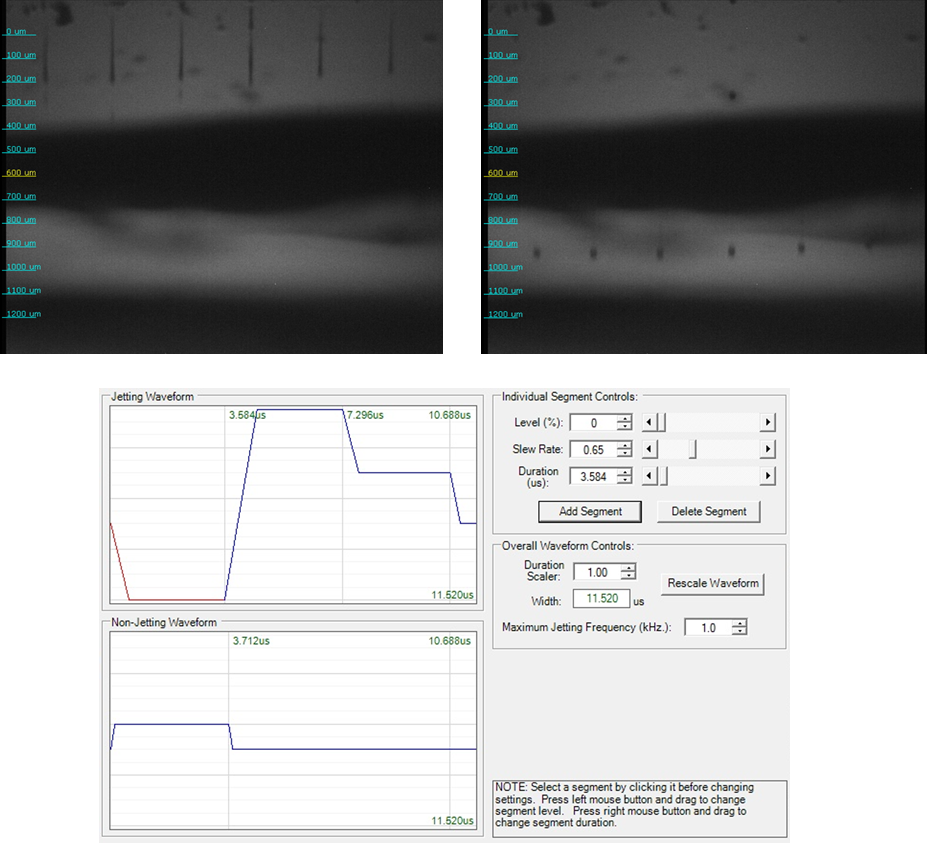


**Figure S25.** Representative drop watcher images and used waveform.

(a)

(b)

(a)

(b)
